# Mediators of change in cognitive behavior therapy and interpersonal psychotherapy for eating disorders: A secondary analysis of a transdiagnostic randomized controlled trial

**DOI:** 10.1002/eat.23390

**Published:** 2020-11-05

**Authors:** Katy Sivyer, Elizabeth Allen, Zafra Cooper, Suzanne Bailey‐Straebler, Marianne E. O'Connor, Christopher G. Fairburn, Rebecca Murphy

**Affiliations:** ^1^ Department of Psychiatry Oxford University Oxford UK; ^2^ Department of Medical Statistics London School of Hygiene and Tropical Medicine London UK; ^3^ Present address: Department of Psychology University of Portsmouth Portsmouth UK; ^4^ Present address: Centre for Clinical and Community Applications of Health Psychology University of Southampton Southampton UK; ^5^ Present address: Department of Psychiatry Yale School of Medicine New Haven Connecticut USA; ^6^ Present address: Department of Psychiatry Weill Cornell Medical College, New York‐Presbyterian Hospital White Plains New York USA

**Keywords:** cognitive behavior therapy, eating disorder, interpersonal psychotherapy, mediator, transdiagnostic, treatment personalization

## Abstract

**Objective:**

Understanding the mechanisms of action of psychological treatments is a key first step in refining and developing more effective treatments. The present study examined hypothesized mediators of change of enhanced cognitive behavior therapy (CBT‐E) and interpersonal psychotherapy for eating disorders (IPT‐ED).

**Method:**

A series of mediation studies were embedded in a randomized controlled trial (RCT) comparing 20 weeks of CBT‐E and IPT‐ED in a transdiagnostic, non‐underweight sample of patients with eating disorders (*N* = 130) consecutively referred to the service. Three hypothesized mediators of change in CBT‐E (regular eating, weighing frequency, and shape checking) and the key hypothesized mediator of IPT‐ED (interpersonal problem severity) were studied.

**Results:**

The data supported regular eating as being a mediator of the effect of CBT‐E on binge‐eating frequency. The findings were inconclusive regarding the role of the other putative mediators of the effects of CBT‐E; and were similarly inconclusive for interpersonal problem severity as a mediator of the effect of IPT‐ED.

**Discussion:**

This research highlights the potential benefits of embedding mediation studies within RCTs to better understand how treatments work. The findings supported the role of regular eating in reducing patients' binge‐eating frequency. Other key hypothesized mediators of CBT‐E and IPT‐ED were not supported, although the data were not inconsistent with them. Key methodological issues to address in future work include the need to capture both behavioral and cognitive processes of change in CBT‐E, and identifying key time points for change in IPT‐ED.

## 1 INTRODUCTION

Understanding how psychological treatments work provides one of the strongest foundations for enhancing their potency (Kazdin & Nock, 2003). Without such understanding, it is unclear whether they work as hypothesized, or whether only some components are key to helping patients recover, while others are redundant. This problem is particularly acute in treatments that have several components and are implemented in a personalized manner.

Both cognitive behavior therapy for eating disorders (CBT‐ED) and interpersonal psychotherapy for eating disorders (IPT‐ED) are evidence‐based treatments (Atwood & Friedman, 2020; NICE, 2017; Norris, Gleaves, & Hutchinson, 2019). They are theoretically distinct and are hypothesized to work in different ways (Murphy, Cooper, Hollon, & Fairburn, 2009). CBT‐ED directly targets specific eating disorder psychopathology and behaviors, whereas IPT‐ED addresses key interpersonal problems thought to be maintaining the eating disorder (Murphy, Straebler, Basden, Cooper, & Fairburn, 2012). Previous research suggests they work in different ways, with IPT‐ED being slower to achieve its effects (Agras, Walsh, Fairburn, Wilson, & Kraemer, 2000; Fairburn et al., 1991, 2015; Fairburn, Jones, Peveler, Hope, & O'Connor, 1993). Although 30–50% of patients achieve good outcomes in both treatments, a significant proportion still have residual psychopathology following treatment (Byrne, Fursland, Allen, & Watson, 2011; Fairburn et al., 2009, 2013, 2015). Further work is therefore needed to make these treatments more potent.

While dismantling studies comparing partial and full versions of CBT‐ED suggest that full versions of CBT‐ED are superior to both behavioral (e.g., Fairburn et al., 1991, 1993) or cognitive elements alone (e.g., Wilson, Rossiter, Kleifield, & Lindholm, 1986), few studies have investigated the role of specific CBT‐ED procedures and the processes they are designed to target (i.e., hypothesized mediators). Research into hypothesized mediators of IPT‐ED has been limited.

This article describes findings from four mediation studies embedded within a transdiagnostic randomized controlled trial (RCT) of non‐underweight patients with eating disorders that compared enhanced cognitive behavior therapy (CBT‐E) for eating disorders, a leading form of CBT‐ED, and IPT‐ED (Fairburn et al., 2015). These mediation studies drew on previous conceptual work outlining hypothesized mediators of both treatments and methodological considerations relevant to their investigation (Murphy et al., 2009). The present research limited its focus to hypothesized mediators of three core CBT‐E procedures (regular eating, weekly weighing, shape checking) and their effects on the specific key eating disorder behavior and psychopathology they target (binge eating, weight concern, shape concern). It also examines what is arguably the key hypothesized mediator of IPT‐ED; interpersonal problem severity. It was hypothesized that;

CBT‐E:

1. decreases binge‐eating frequency through increasing **regular eating**;
2. decreases weight concern by decreasing **weighing frequency**;
3. decreases shape concern by decreasing **shape checking**;

IPT‐ED:

4. decreases overall eating disorder psychopathology by decreasing **interpersonal problem severity**.

## 2 METHOD

### 2.1 Ethics

Ethical approval was obtained from the National Research Ethics Service Oxfordshire Research Ethics Committee C (REF: 06/Q1606/82).

### 2.2 Design

Four mediation studies were embedded within an RCT comparing CBT‐E (*N* = 65) and IPT‐ED (*N* = 65) (Current controlled trials: ISRCTN 15562271). There was a closed 60‐week follow‐up during which patients received no other treatment unless clinically essential (see Fairburn et al., 2015).

### 2.3 Sample

One hundred and thirty adult patients (98% female; 95% white; mean age 26 years) were recruited through consecutive referrals to an eating disorder clinic. Patients were assessed for DSM‐IV eating disorder diagnosis (American Psychiatric Association, 1994), consented and randomized. Participants had a body mass index between 17.5 and 39.9 (inclusive), and had not previously received CBT‐E or IPT‐ED. Fifty‐three patients (41%) met diagnostic criteria for bulimia nervosa and 77 (59%) met criteria for eating disorder not otherwise specified, of whom eight (10%) had binge‐eating disorder.

### 2.4 Treatments

In both treatments patients attended one preparatory session (90 min), followed by 20 50‐min individual sessions over 20 weeks and a review session 20 weeks after treatment had ended.

#### 2.4.1 Enhanced cognitive behavior therapy—Focused version

CBT‐E (Fairburn, Cooper, Shafran, et al., 2008) has multiple procedures that are implemented sequentially in a flexible manner. Each procedure directly targets one or more specific features hypothesized to maintain the eating disorder. Key procedures in the first two stages of treatment are “regular eating” and “weekly weighing.” “Regular eating” addresses one form of dietary restraint; delayed eating, which is hypothesized to maintain binge eating. It involves establishing a regular pattern of eating of three meals and two snacks a day (without changing the quantity or variety of food). “Weekly weighing” targets frequent weighing, which is hypothesized to maintain preoccupation and overevaluation of weight. It involves in‐session weekly weighing that is jointly interpreted with the therapist. In stage three, treatment procedures are individualized in accordance with the patient's personal formulation of the factors maintaining their psychopathology. For most patients, “shape checking” is a key procedure that addresses frequent shape checking, which is hypothesized to maintain preoccupation and overevaluation of shape. It involves self‐monitoring shape checking behaviors, evaluating their utility, and reducing their frequency.

#### 2.4.2 Interpersonal psychotherapy for eating disorders

IPT‐ED (Fairburn, 1992, 1997; Murphy et al., 2012) is derived from IPT for depression (Klerman, Weissman, Rounsaville, & Chevron, 1984) and closely resembles it. Treatment targets the key interpersonal problem(s) thought to be maintaining the patient's eating disorder, focusing on one or two of the following key problem areas; interpersonal role disputes, role transitions, grief, interpersonal deficits, or life goals. Identified problem areas are examined in detail and ways to resolve them are discussed. Treatment is conceptualized as a unitary intervention to reduce eating disorder psychopathology, rather than a collection of separate procedures (Murphy et al., 2009).

### 2.5 Therapists

The same therapists delivered both treatments. All received 6 months training in CBT‐E and IPT‐ED before starting the trial and were supervised weekly thereafter. Treatment sessions were recorded and audited to ensure treatment fidelity. A random audit confirmed high levels of fidelity (Fairburn et al., 2015).

### 2.6 Procedure and measures

**FIGURE 1:**
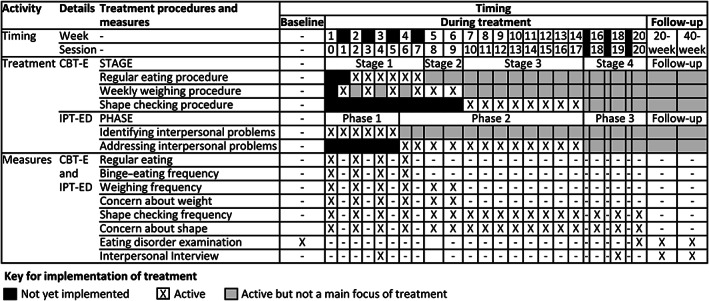
Treatment and measurement timings in enhanced cognitive behavior therapy (CBT‐E) and interpersonal psychotherapy for eating disorders (IPT‐ED)

Each hypothesis was examined separately, with careful attention to the timing of measurement so as to detect change following implementation of treatment and to avoid confounding with other treatment procedures (Murphy et al., 2009). In CBT‐E this focused on the specific timing of the implementation of each treatment procedure of interest while in IPT‐ED treatment was assessed in a unitary fashion over a longer period (see Figure 1) because existing evidence shows that IPT‐ED is slower to achieve its effects (Agras et al., 2000; Fairburn et al., 1991, 1993, 2015). Measures of all hypothesized mediators and outcomes were assessed in both treatments (see below). Weekly measures of eating disorder symptoms and behaviors were based on the Eating Disorder Examination (EDE; 16.0) clinical interview (Fairburn, Cooper, & O'Connor, 2008), which was administered at baseline, end of treatment, and 20‐week, 40‐week, and 60‐week follow‐up in the main trial (Fairburn et al., 2015).

#### 2.6.1 Hypothesis 1: Regular eating and binge‐eating frequency

Regular eating and binge‐eating frequency were assessed during the first 4 weeks of treatment, when “regular eating” is a key focus of CBT‐E and most changes attributable to this procedure are thought to occur (Fairburn, Cooper, Shafran, et al., 2008).

Independent blind raters used patients' self‐monitoring records to assess the following for each patient each week:

- **Regular eating (hypothesized mediator)**, patient's adherence to a daily eating pattern of three meals plus two or three snacks, rated on a scale of “0” (absence of regular eating) to “6” (marked adherence to regular eating).
- **Binge‐eating frequency (outcome)**, number of objective binge‐eating episodes, defined as eating a substantially larger amount of food than most people would eat during a 2‐hr period, with sense of control not assessed as the monitoring record captured only whether food intake was considered “excessive.”

Records were first rated separately by two raters, who then consulted to agree the final rating. Since it was hypothesized that the effect of the “regular eating” procedure might differ between patients who did and did not binge eat at baseline, this was assessed as a moderator (based on baseline EDE).

#### 2.6.2 Hypothesis 2: Weighing frequency and concern about weight

Weighing frequency and concern about weight were assessed for the first 6 weeks of treatment, when “weekly weighing” is a key focus of treatment and before other treatment procedures are introduced that might impact on these variables (e.g., “shape checking”, or other Stage 3 procedures that focus on increasing variety of food intake).

Patients self‐reported each week:

- **Weighing frequency (hypothesized mediator)**, the number of times they had weighed themselves.
- **Concern about weight (outcome)** on a scale of “0” (not at all) to “6” (markedly).

Since it was hypothesized that the effect of the weekly weighing procedure might differ between patients who at baseline did and did not frequently weigh themselves, this was assessed as a moderator (using a once per week cut‐off, the frequency of weighing in‐session in CBT‐E).

#### 2.6.3 Hypothesis 3: Shape checking and concern about shape

Shape checking and concern about shape were assessed throughout treatment as the use and timing of this procedure was personalized to the patient. Only patients who received this procedure were included in the “intervention group,” with the control group comprising both CBT‐E patients who did not receive the procedure and IPT‐ED patients.

Patients self‐reported each week:

- **Shape checking (hypothesized mediator)**, how often they had actively checked their body shape or size in; (a) mirrors, and (b) by pinching or measuring their body, rated on a scale of “1” (not at all) to “5” (many times a day). These were averaged into a single score.
- **Concern about shape (outcome)** rated on a scale of “0” (not at all) to “6” (markedly).

#### 2.6.4 Hypothesis 4: Interpersonal problem severity and eating disorder psychopathology

Interpersonal problem severity and eating disorder psychopathology were assessed at the beginning and end of treatment, and 20 and 40 weeks after treatment. This timeframe was chosen because it was hypothesized that a reduction in problem severity would take considerable time and that in turn this would lead to a progressive improvement in psychopathology (Murphy et al., 2009).

Three independent, blind raters conducted semistructured interviews to assess:

- **Interpersonal problem severity (hypothesized mediator)**, the severity of the patient's key interpersonal problem (in IPT‐ED this was the problem targeted in treatment), rated on a scale of “0” (no problem—no or minor difficulties, no impairment) to “6” (marked problem—difficulties occurring most of the time, substantial impairment).
- **Eating disorder psychopathology (outcome)**, assessed using the global score from the EDE.

Assessors were trained and supervised by MO'C, an expert in the EDE.

### 2.7 Data analysis

The hypothesized relationships between treatment, the hypothesized mediator and outcome were assessed using statistical mediation. A two‐stage analytic strategy was used as described in Sivyer et al. (2020).

Stage 1 explored the effect that treatment had on change in the hypothesized mediator and outcome over time using multilevel modeling (Krull & MacKinnon, 1999, 2001). Random effects were fitted at the patient level and on time. Treatment, time, relevant predictors and moderators, and the interactions between treatment and time, treatment and the moderator, and treatment and the hypothesized mediator were included as independent variables. For personalized treatment procedures the week of implementation of the personalized procedure was also included as an independent variable (Sivyer et al., 2020).

Stage 2 explored the relationships between treatment, the hypothesized mediator, and the outcome over time using autoregressive structural equation modeling (Cole & Maxwell, 2003), with relationships specified for treatment predicting the hypothesized mediator and outcome, and the hypothesized mediator predicting the outcome.

The following effects were assessed at each time point:

- The “indirect effect” of treatment on the outcome (i.e., the effect of treatment achieved via the hypothesized mediator and, at later time points, through change achieved earlier in treatment).
- The “total effect” of treatment on the outcome (i.e., the overall effect of treatment on the outcome, through both its direct effect on the outcome and its indirect effect through the hypothesized mediator and change achieved earlier in treatment).

For personalized treatment procedures, the model examined the first 6 weeks of implementation of the personalized treatment procedure compared to a similar time period in the control group (based on the average week of implementation in the intervention group) (Sivyer et al., 2020). Model fit was considered acceptable where; chi‐square *p* > .05, comparative fit index (CFI) > .95, and root mean squared error of approximation (RMSEA) <0.08 with *p*close <.05 (Kline, 2011). Standardized betas were used as a measure of effect size for both indirect and total effects. Where the results were consistent with the hypothesized model, a second, reversed model was run in which the outcome predicted the hypothesized mediator to verify the direction of these relationships.

Statistical mediation was concluded only if the results across both stages and the models within them were consistent regarding the hypothesized mediator mediating the effect of treatment on the outcome. As the analyses were investigating different, clearly specified hypotheses, correction for multiple testing was not applied (Perneger, 1998; Rothman, 1990). All models were bootstrapped based on 1,000 resamples to correct for non‐normality in the data. Analyses were undertaken in Stata 13 (StataCorp, 2013). Multilevel modeling used restricted maximum likelihood estimation. Autoregressive structural equation modeling used full information maximum likelihood estimation to account for missing data. More detailed information about model specification can be found in Sivyer et al. (2020).

## 3 RESULTS

**FIGURE 2:**
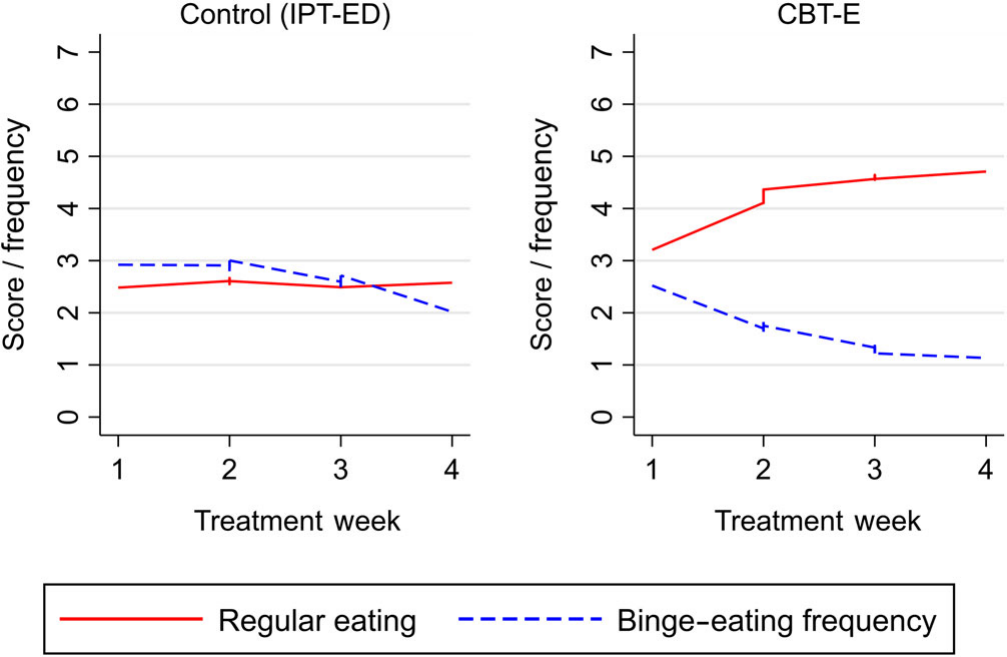
Smoothed line graphs comparing the observed means of regular eating and binge‐eating frequency in Enhanced Cognitive Behavior Therapy (CBT‐E) and Interpersonal Psychotherapy for Eating Disorders (IPT‐ED) [Color figure can be viewed at wileyonlinelibrary.com]

**FIGURE 3:**
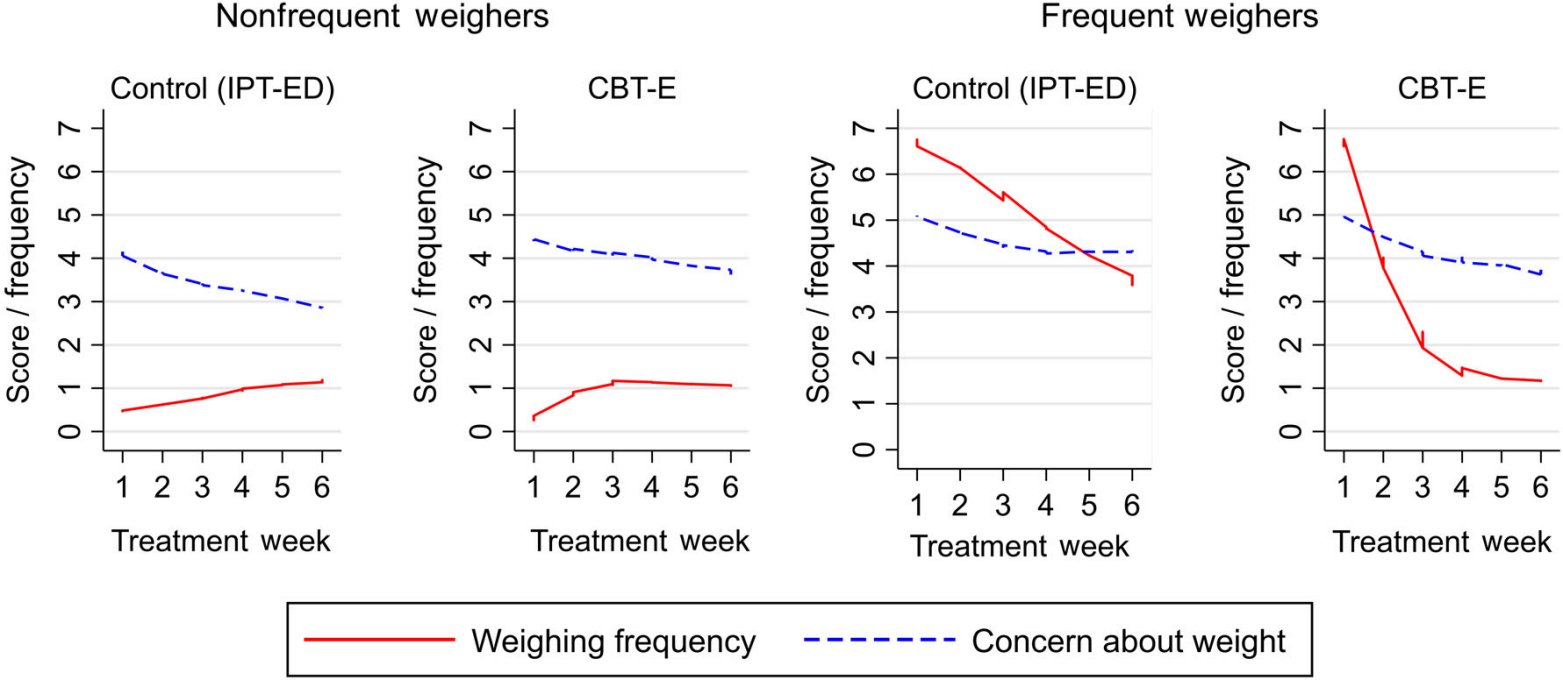
Smoothed line graphs comparing the observed means of weighing frequency and concern about weight in frequent and nonfrequent weighers in Enhanced Cognitive Behavior Therapy (CBT‐E) and Interpersonal Psychotherapy for Eating Disorders (IPT‐ED) [Color figure can be viewed at wileyonlinelibrary.com]

**FIGURE 4:**
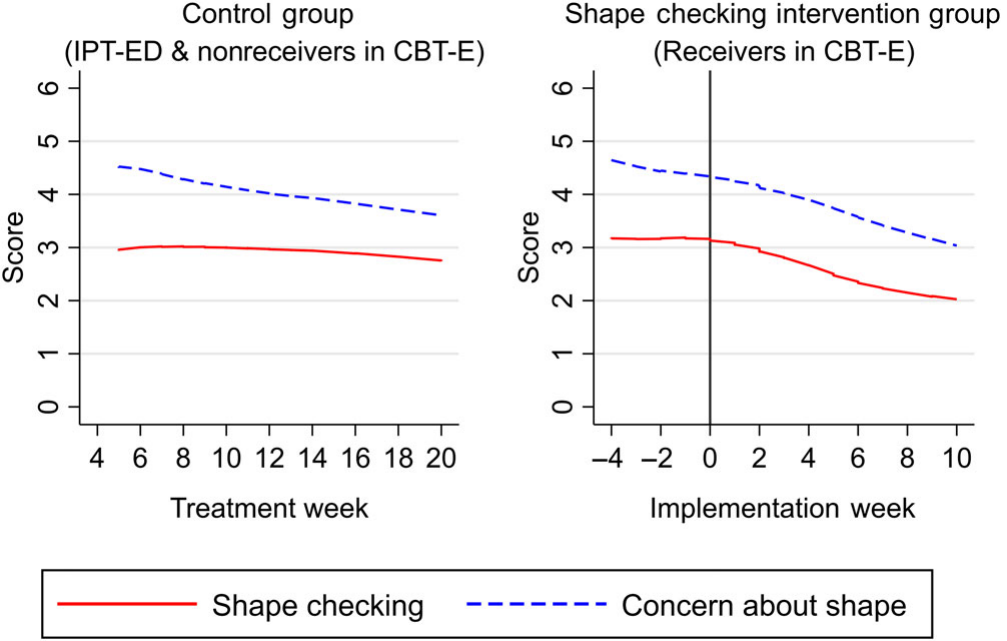
Smoothed line graphs comparing the observed means of shape checking and concern about shape in patients who received the shape checking procedure in Enhanced Cognitive Behavior Therapy (CBT‐E) and patients who did not (patients in Interpersonal Psychotherapy for Eating Disorders (IPT‐ED) and nonreceivers in CBT‐E) [Color figure can be viewed at wileyonlinelibrary.com]

**FIGURE 5:**
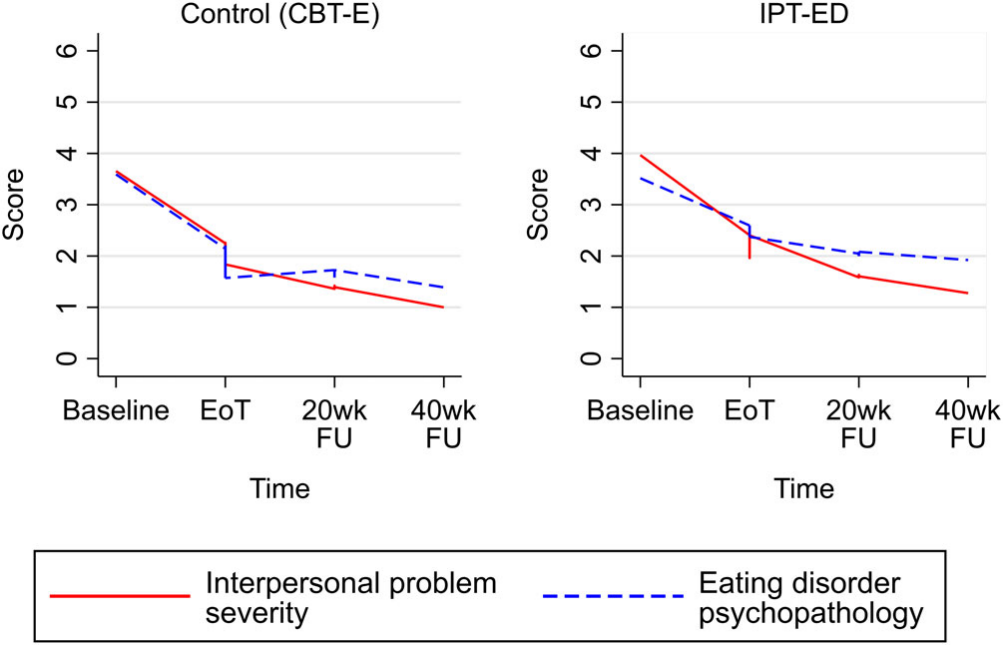
Smoothed line graphs comparing the observed means of interpersonal problem severity and eating disorder psychopathology in Interpersonal Psychotherapy for Eating Disorders (IPT‐ED) and Enhanced Cognitive Behavior Therapy (CBT‐E). EoT, end of treatment; 20wk FU, 20‐week follow‐up; 40wk FU, 40‐week follow‐up [Color figure can be viewed at wileyonlinelibrary.com]

Baseline descriptives and measure validity are reported in Table 1, with change over time shown in smoothed mean line graphs for each hypothesis (Figures 2, 3, 4, 5). Key model coefficients for each hypothesis are reported in Table 2. Full model outputs for all multilevel modeling and structural equational modeling analyses are included in the Supporting Information. The main results are summarized below.

**TABLE 1:** Baseline descriptives and assessments of measure validity

Variables	Enhanced cognitive behavior therapy (CBT‐E)			Interpersonal psychotherapy for eating disorders (IPT‐ED)			Measure validity of unvalidated measures
*Hypothesis 1: Regular eating and binge‐eating frequency*							
	**Mean**	***SD***	**Total *N***	**Mean**	***SD***	**Total *N***	Regular eating score and EDE rating of adherence at baseline; *r* = .47
Regular eating score	3.21	1.09	58	2.48	1.47	58	
EDE eating pattern rating of adherence to a regular pattern of eating in previous 4 weeks	3.34	0.99	65	3.13	1.30	65	
	***n***	**%**	**Total *N***	***n***	**%**	**Total *N***	
Regular eating score of “pretty good” or “marked” adherence (≥5)	4	7%	58	3	5%	58	
EDE eating pattern rating of “almost adherent” or “adherent” to a regular pattern of eating in previous 4 weeks (≥5)	5	8%	65	5	8%	65	
	**Median**	**IQR**	**Total *N***	**Median**	**IQR**	**Total *N***	Binge‐eating frequency and EDE objective bulimic episodes at baseline; *r* = .45
Binge‐eating frequency	1.40	0.00, 4.69	58	1.19	0.00, 5.25	59	
For those who binge eat^a^	1.58	0.00, 4.69	50	2.80	0.00, 7.00	46	
EDE objective episodes of bulimia (weekly rate over previous 4 weeks)	2.75	1.00, 7.00	65	3.50	0.75, 7.00	65	
For those who binge eat^a^	3.75	2.00, 8.00	54	5,25	1.50, 9.00	51	
	***n***	**%**	**Total *N***	***n***	**%**	**Total *N***	
Binge‐eating present	36	62%	58	32	54%	59	
EDE rating binge‐eating present in previous 4 weeks	54	83%	65	51	78%	65	
*Hypothesis 2: Weighing frequency and concern about weight*							
	**Median**	**IQR**	**Total *N***	**Median**	**IQR**	**Total *N***	Weighing frequency and EDE weighing frequency at baseline; *r* = .76
Weighing frequency	0.00	0.00, 2.50	64	1.00	0.00, 4.00	65	
In frequent weighers (weighing >1 a week)^a^, ^b^	5.00	1.50, 8.00	24	6.00	3.00, 7.00	27	
EDE weighing frequency	0.00	0.00, 2.50	65	0.50	0,00, 2.50	65	
In frequent weighers (weighing >1 a week)^a^, ^b^	5.00	2.13, 7.00	24	4.00	1.75, 7.00	27	
	***n***	**%**	**Total *N***	***n***	**%**	**Total *N***	
Weighing frequency of >1 a week	20	31%	64	29	45%	65	
EDE weighing frequency >1 a week in previous 4 weeks	24	37%	65	27	42%	65	
EDE rating weighing avoidance in previous 4 weeks	6	9%	65	5	8%	65	
	**Mean**	***SD***	**Total *N***	**Mean**	***SD***	**Total *N***	Concern about weight score and EDE weight concern subscale at baseline; *r* = .67
Concern about weight score	4.77	1.57	64	4.58	1.71	65	
EDE weight concern subscale score	3.77	1.31	65	3.47	1.51	65	
*Hypothesis 3: Shape checking frequency and concern about shape*							
	**Mean**	***SD***	**Total *N***	**Mean**	***SD***	**Total *N***	Shape checking score and EDE rating of shape vigilance at baseline; *r* = .67 Internal reliability for shape checking score at each time point; *r* = .56–.82
Shape checking score	3.28	1.28	64	3.13	1.23	65	
EDE shape vigilance rating	3.57	2.56	65	3.82	2.42	65	
	***n***	**%**	**Total *N***	***n***	**%**	**Total *N***	
Shape checking score of daily or more (≥3)	43	67%	64	39	60%	65	
Shape checking score of no checking at all (1)	6	9%	64	3	5%	65	
EDE shape vigilance rating of daily in previous 4 weeks (6)	30	46%	65	29	44%	65	
EDE rating of zero shape vigilance in previous 4 weeks (0)	14	22%	65	13	20%	65	
	**Mean**	***SD***	**Total *N***	**Mean**	***SD***	**Total *N***	Concern about shape score and EDE shape concern subscale at baseline; *r* = .70
Concern about shape score	5.22	1.46	64	5.31	1.03	65	
EDE shape concern subscale score	4.08	1.38	65	4.03	1.32	65	
*Hypothesis 4: Interpersonal problem severity and eating disorder psychopathology*							
	**Mean**	***SD***	**Total *N***	**Mean**	***SD***	**Total *N***	Weighted Kappas (Landis & Koch, 1977) assessing agreement between raters; Kappa = .79–.93.
Interpersonal problem severity score	3.66	1.05	61	3.97	1.18	63	
	***n***	**%**	**Total *N***	***n***	**%**	**Total *N***	
Key interpersonal problem							
Interpersonal deficits	30	48%	62	28	44%	63	
Interpersonal role disputes	20	32%	62	28	44%	63	
Role transition	9	15%	62	6	10%	63	
Life goals	3	5%	62	0	0%	63	
Grief	0	0%	62	1	2%	63	
	**Mean**	***SD***	**Total *N***	**Mean**	***SD***	**Total *N***	N/A—validated measure
EDE global eating disorder psychopathology score	3.59	1.01	65	3.52	1.05	65	

*Note*: EDE, Eating Disorder Examination.

a Group membership based on baseline EDE.

b Excluding patients exhibiting weighing avoidance.

**TABLE 2:** Key coefficients of multilevel (MLM) and structural equation (SEM) models examining the impact of treatment on the hypothesized mediator and outcome, and the indirect and total effects of treatment on the outcome for all hypotheses in the first week following implementation and the last time point studied

Statistical model^a^	Treatment effects	*B*	*SE*	Bias‐corrected confidence intervals		*p*	*β*	Consistent with mediation?
				Lower	Upper			
*Hypothesis 1: Regular eating as a mediator of the effect of CBT‐E on binge‐eating frequency (CBT‐E* vs. *IPT‐ED)*								
MLM	Effect of CBT‐E on regular eating each week^b^	1.12	0.21	0.68	1.51	<.001	—	Yes
	Effect of CBT‐E on binge‐eating frequency each week^b^	−1.40	0.51	−2.47	−0.47	.007	—	Yes
AR SEM	Indirect effect of CBT‐E at Week 2 of treatment	−0.86	0.37	−1.65	−0.27	.02	−.14	Yes
	Indirect effect of CBT‐E at Week 4 of treatment	−1.40	0.55	−2.64	−0.48	.01	−.27	Yes
	Total effect of CBT‐E at Week 4 of treatment	−0.45	0.42	−1.34	0.31	.29	−.09	N/A
	Fit indices	*Χ* ^2^ (23, *N* = 130) = 23.369, *p* = .439, RMSEA = .011, *p* = .786, CFI = .999						
*Hypothesis 2: Weighing frequency as a mediator of the effect of CBT‐E on concern about weight (CBT‐E vs. IPT‐ED, frequent vs. nonfrequent weighers)*								
MLM	Effect of CBT‐E on weighing frequency each week (nonfrequent weighers^c^)	0.48	0.28	−0.05	1.06	.09	—	No
	Effect of CBT‐E on weighing frequency each week (frequent weighers^b^ ^,^ ^c^)	−3.25	0.91	−5.00	−1.56	.001	—	Yes
	Effect of IPT‐ED on weighing frequency each week (frequent weighers^c^)	−0.51	0.48	−1.40	0.46	.29	—	No
	Effect of CBT‐E on concern about weight each week	0.04	0.05	−0.06	0.13	.43	—	No
AR SEM	Indirect effect of CBT‐E at Week 2 of treatment (nonfrequent weighers)	0.02	0.05	−0.02	0.18	.63	.01	No
	Indirect effect of CBT‐E at Week 6 of treatment (nonfrequent weighers)	−0.16	0.07	−0.98	0.57	.70	−.04	No
	Total effect of CBT‐E at Week 6 of treatment (nonfrequent weighers)	0.10	0.21	−0.31	0.51	.75	.03	N/A
	Indirect effect of CBT‐E at Week 2 of treatment (frequent weighers)	−0.13	0.14	−0.45	0.10	.36	.10	Trend
	Indirect effect of CBT‐E at Week 6 of treatment (frequent weighers)	−0.27	0.23	−0.71	0.17	.24	.17	Trend
	Total effect of CBT‐E at Week 6 of treatment (frequent weighers)	−0.27	0.23	−0.71	0.17	.24	.17	N/A
	Fit indices	*Χ* ^2^ (77, *N* = 130) = 92.976, *p* = .104, RMSEA = .040; *p* = .703, CFI = .990						
*Hypothesis 3: Shape checking frequency as a mediator of the effect of CBT‐E on concern about shape in the shape checking intervention group vs. control group (including patients in IPT‐ED and those in CBT‐E who did not receive the shape checking intervention)*								
MLM	Effect of shape checking intervention on shape checking frequency each week	−0.18	0.05	−0.26	−0.08	<.001	—	Yes
	Effect of shape checking intervention on concern about shape each week	−0.09	0.03	−0.14	−0.03	.002	—	Yes
AR SEM	Indirect effect of shape checking intervention at Week 1 of implementation	0.14	0.08	0.03	0.36	.08	.04	Yes—Based on bias‐corrected confidence interval, but in opposite direction
	Indirect effect of shape checking intervention at Week 6 of implementation	−0.61	0.40	−1.45	0.18	.13	−.16	Trend
	Total effect of shape checking intervention at Week 6 of treatment	−0.40	0.36	−1.10	0.34	.27	−.11	N/A
	Fit indices	*Χ* ^2^ (104, *N* = 130) = 121.986, *p* = .110; RMSEA = .036, *p* = .801, CFI = .985						
*Hypothesis 4: Interpersonal problem severity as a mediator of the effect of IPT‐ED on global eating disorder psychopathology (IPT‐ED vs. CBT‐E)*								
MLM	Effect of IPT‐ED on interpersonal problem severity during treatment and follow‐up	−0.01	0.13	−0.27	0.28	.93	—	No
	Effect of IPT‐ED on global eating disorder psychopathology during treatment and follow‐up^b^	0.59	0.28	0.07	1.12	.03	—	No
AR SEM	Indirect effect of IPT‐ED at week the end of treatment	−0.05	0.10	−0.30	0.12	.57	−.02	No
	Indirect effect of IPT‐ED at 40‐week follow‐up	0.13	0.16	−0.15	0.50	.41	.06	No
	Total effect of CBT‐E at 40‐week follow‐up	0.41	0.22	−0.04	0.83	.06	.17	N/A
	Fit indices	*Χ* ^2^ (20, *N* = 130) = 17.145, *p* = .644, RMSEA = .000; *p* = .887, CFI = 1.000						

*Abbreviations*: AR SEM, autoregressive structural equation modeling; *B*, unstandardized estimate; CBT‐E, enhanced cognitive behavior therapy; IPT‐ED, interpersonal psychotherapy for eating disorders; MLM, multilevel modeling; *SE*, standard error; *β*, standardized estimate.

^a^ All models controlling for relevant predictors and moderators. SEM models also controlling for shared measurement variance. Confidence intervals are bias‐corrected based on 1,000 bootstrap resamples. *p* values based on uncorrected 95% confidence intervals. Indirect and total effects of treatment incorporate transmission of treatment effects via autoregressive paths.

^b^ Indicates the presence of a nonlinear trend suggesting that effects plateau toward the end of the treatment period studied.

^c^ Indicates that this was compared to nonfrequent weighers in IPT‐ED.

### 3.1 Hypothesis 1: Regular eating and binge‐eating frequency

#### 3.1.1 Multilevel models: Change during treatment

Regular eating showed a greater weekly increase in CBT‐E compared to IPT‐ED, stabilizing around Week 3 of treatment. There was no change in regular eating across the first 4 weeks of IPT‐ED. Binge‐eating frequency decreased in both treatments; however, it declined earlier in CBT‐E compared to IPT‐ED (Week 2 vs. Week 4, respectively). In CBT‐E, the rate of decline slowed around Week 3. In CBT‐E, change in the hypothesized mediator and outcome occurred simultaneously, with decreases in binge‐eating frequency mirroring the increases in regular eating (Figure 2).

The effect of CBT‐E and IPT‐ED on regular eating and binge‐eating frequency did not differ between patients who were binge eating at baseline and those who were not, suggesting that binge‐eating status was not a moderator.

#### 3.1.2 Autoregressive structural equation model: Indirect effects

There was a negative relationship between regular eating and binge‐eating frequency in all treatment weeks, including baseline; however, the relationship over time was complex. Within the same treatment week greater regular eating was associated with lower binge‐eating frequency as hypothesized, but between treatment weeks a positive relationship was observed, with higher levels of regular eating associated with increased binge eating the following week. Overall, there was evidence of an indirect effect of CBT‐E decreasing binge‐eating frequency via regular eating. This effect was found immediately following the implementation of the “regular eating” procedure in Week 2 of treatment, and was still present at Week 4. The reversed model assessing whether change in regular eating was better explained by the direct effect that CBT‐E had on binge‐eating frequency, rather than the other way around, supported regular eating as a mediator.

### 3.2 Hypothesis 2: Weighing frequency and concern about weight

#### 3.2.1 Multilevel models: Change during treatment

Weighing frequency differed between frequent and nonfrequent weighers in CBT‐E and IPT‐ED at baseline and during treatment. Weighing frequency decreased more rapidly in frequent weighers in CBT‐E compared to nonfrequent weighers and compared to frequent weighers in IPT‐ED. Change in weighing frequency in frequent weighers in CBT‐E stabilized by Week 3. Weighing frequency did not change significantly during the first 6 weeks of treatment in nonfrequent weighers in both treatments or in frequent weighers in IPT‐ED.

Concern about weight decreased at a similar rate in both treatments in frequent and nonfrequent weighers; −0.36, [−0.52, −0.22]. This rate of change slowed by Week 6. Change in the hypothesized mediator appeared to have little observable relationship to change in the outcome in either treatment (Figure 3).

#### 3.2.2 Autoregressive structural equation model: Indirect effects

There was a positive relationship between weighing frequency and concern about weight at baseline in the direction hypothesized, with more frequent weighing associated with increased concern about weight. However, the relationship between these variables was inconsistent during treatment. Although there was a trend toward CBT‐E decreasing concern about weight via decreased weighing frequency in frequent weighers, this did not reach statistical significance in either frequent or nonfrequent weighers at any week, despite CBT‐E having a greater effect in decreasing weighing frequency.

### 3.3 Hypothesis 3: Shape checking and concern about shape

#### 3.3.1 Multilevel models: Change during treatment

The shape checking procedure was used with 53 patients in CBT‐E (82%). On average, it was implemented in Treatment Week 8 (mean = 7.66, *SD* = 2.01).

Prior to its implementation, shape checking initially remained stable in both CBT‐E and IPT‐ED. As treatment progressed patients in CBT‐E gradually started to increase their shape checking. However, following implementation of the shape checking procedure, those patients in CBT‐E who received the intervention began to decrease their shape checking over time.

Concern about shape decreased in both IPT‐ED and CBT‐E. However, for patients in the shape checking intervention group, concern about weight decreased even further following implementation of the shape checking procedure. Change in concern about shape slowed toward the end of treatment across all groups. Shape checking and concern about shape had a similar trajectory in those who received the shape checking procedure (Figure 4).

#### 3.3.2 Autoregressive structural equation model: Indirect effects

There was a positive relationship between shape checking and concern about shape at baseline as hypothesized, with higher shape checking scores associated with increased concern about shape. However, the relationship between these variables was inconsistent during treatment. There was a trend toward an indirect effect of the shape checking procedure decreasing concern about shape via decreased shape checking; however, this did not reach statistical significance. Instead, there was a statistically significant indirect effect for the shape checking procedure temporarily increasing concern about shape via increased shape checking immediately following implementation of the shape checking procedure (Week 1 of the intervention). However, the effect size was negligible.

### 3.4 Hypothesis 4: Interpersonal problem severity and eating disorder psychopathology

#### 3.4.1 Multilevel models: Change during treatment

Interpersonal problem severity decreased in both IPT‐ED and CBT‐E at a similar rate; −2.02, [−2.41, −1.61]. The rate of change plateaued during follow‐up, with little further change occurring after 20‐week follow‐up. This suggests that most change occurred during treatment. Eating disorder psychopathology decreased in both treatments but remained higher in IPT‐ED compared to CBT‐E, although this difference started to narrow during follow‐up. Interpersonal problem severity and eating disorder psychopathology had similar trajectories in both treatments (Figure 5).

#### 3.4.2 Autoregressive structural equation model: Indirect effects

Overall, there was a positive relationship between interpersonal problem severity and eating disorder psychopathology throughout treatment, except at 20‐week follow‐up. There was evidence of an indirect effect of IPT‐ED increasing eating disorder psychopathology at 20‐week follow‐up. This was unlikely due to the effect of IPT‐ED on eating disorder psychopathology via interpersonal problem severity as there were no differences between treatments in terms of interpersonal problem severity during this period. Instead, this likely reflects that the difference between treatments in eating disorder psychopathology at the end of treatment was carried over into 20‐week follow‐up. This disappeared as the gap between the two treatments narrowed.

## 4 DISCUSSION

This study investigated hypothesized mechanisms of CBT‐E and IPT‐ED using data from an RCT. Three hypothesized mediators of CBT‐E (regular eating, weighing frequency, and shape checking) and the key hypothesized mediator of IPT‐ED (interpersonal problem severity) were examined. Of the three hypothesized mediators of CBT‐E examined, only regular eating was consistent with it being a mediator of the effect of CBT‐E on binge‐eating frequency. The findings were inconclusive for the other two hypothesized mediators of CBT‐E, and for the hypothesized mediator of IPT‐ED.

Regular eating has consistently been associated with decreased frequency of binge eating (Ellison et al., 2016; Shah, Passi, Bryson, & Agras, 2005; Waller, Evans, & Pugh, 2013; Wilson, Fairburn, Agras, Walsh, & Kraemer, 2002; Zendegui, West, & Zandberg, 2014). However, this is the first study to assess change in both variables on a weekly basis. It is unlikely that these findings are due to patients increasing food quantity or variety as patients were advised to change only their eating pattern (see Fairburn, Cooper, Shafran, et al., 2008). Nonetheless, some patients may have made further dietary changes.

Overall the results supported the hypothesized negative relationship between regular eating and binge‐eating frequency during the first 4 weeks of treatment and within the same treatment week; however, there was also a positive relationship between regular eating and binge‐eating frequency between treatment weeks. This may reflect lapses in regular eating and binge eating between treatment weeks, falsely making it look like high levels of regular eating the previous week were associated with higher levels of binge eating the following week. Further research should explore potential lapses during treatment in binge eating, possibly using an ecological momentary assessment design with more frequent measurement of these behaviors.

Binge‐eating frequency decreased at different rates in the two treatments. In IPT‐ED change in binge‐eating frequency appeared to occur independently of change in regular eating, which remained stable during the period studied. This suggests that the effect of IPT‐ED on binge‐eating frequency may be mediated via a different process, as would be hypothesized by the different models underpinning these treatments.

Only one study has investigated the role of frequent weight and shape checking in CBT‐E, which, consistent with the CBT‐E model, found that decreased body checking was associated with decreased concern about shape in inpatients with anorexia nervosa (Calugi, El Ghoch, & Dalle Grave, 2017). The current research did not replicate these findings. This may be due to sample differences (inpatients vs. outpatients, underweight vs. non‐underweight), or issues with statistical power.

A temporary increase in concern about shape has been suggested as a potential side‐effect of monitoring shape checking (Fairburn, Cooper, Shafran, et al., 2008). Many patients are unaware of how frequently they check their shape and bringing these behaviors into conscious awareness can be distressing. The temporary increase in concern about shape found in this study is consistent with this. Raised awareness of these behaviors may also explain the increased self‐reporting in shape checking. Although differences might be expected between patients who shape check and those who do not, in general patients self‐reported high levels of shape checking at baseline, with 63% (*N* = 82) checking their shape at least daily. Very few did not check their shape (*N* = 9; 7%). However, more research is needed to examine other relevant behaviors such as shape avoidance.

Both “weekly weighing” and “shape checking” are complex procedures addressing behaviors and cognitions. This study measured only the behavioral aspect of these procedures (i.e., frequency of weighing or shape checking). As such, it may have missed important cognitive changes affecting concern about weight and shape. For example, more frequent weighing would not necessarily increase concern about weight in the presence of more benign interpretations of weight fluctuations. Future work should consider assessing both cognitive and behavioral processes associated with these procedures, and other hypothesized mediators (e.g., managing moods/events).

As there was little change in interpersonal problem severity and eating disorder psychopathology in both treatments during follow‐up, it was difficult to draw conclusions regarding the mediational relationship between these variables. There has been relatively little research into the mechanisms of IPT‐ED, however, previous research has identified that interpersonal problems improve in both CBT‐ED and IPT‐ED (Fairburn et al., 1993; Wilfley et al., 2002). It is unclear whether this is the result of improving interpersonal problem severity, improving eating disorder psychopathology, or both. The fact that both IPT‐ED and CBT‐E improve interpersonal problem severity, whether directly or indirectly, may explain why examining IPT‐ED as a secondary treatment for nonresponders to CBT‐ED has not had much success (Mitchell et al., 2002). Further work should examine change in interpersonal problem severity and eating disorder psychopathology during treatment, rather than at follow‐up to gain a better understanding of these processes and use more frequent measurement.

### 4.1 Strengths and limitations

This research used some unvalidated measures. As such little is known their psychometric properties, although preliminary analyses suggest moderate‐strong correlations with equivalent items on the EDE at baseline and interrater reliability for the interpersonal interview was good. Binge‐eating frequency did not assess sense of loss of control, and hence “objective overeating” was used as a proxy for objective binge eating.

The sample size (*N* = 130) was relatively small for the structural equation models used. It is likely that tests of the indirect effect were underpowered, which is a common problem in such analyses (MacKinnon, Lockwood, Hoffman, West, & Sheets, 2002). However, there is a lack of definitive research into the sample size requirements for longitudinal statistical mediation (Cole & Maxwell, 2003; Little, 2013). Although personalization is a clinical strength of these treatments, there has been little research regarding how to examine mediational processes under such conditions. Further work is needed to identify the best methods.

Although research suggests that early change during treatment consistently predicts better outcomes (Linardon, de la Piedad Garcia, & Brennan, 2017), this research did not examine whether change in the hypothesized mediators was maintained or was related to longer‐term outcomes.

A key strength was that the mediation studies were embedded within a carefully designed RCT, a design that has been underutilized in assessing how treatments work (Dunn et al., 2015; Kraemer, Wilson, Fairburn, & Agras, 2002). Treatment fidelity was high (Fairburn et al., 2015). Randomization ensured that the analyses were less likely to be influenced by confounders, at least in the case of the relationship between treatment and mediator, and treatment and outcome for the nonpersonalized elements of the treatments. The hypothesized mediator and outcome variables were both measured throughout the period during which change is likely to occur. This was planned a priori to deal with the challenge of ensuring that changes in the hypothesized mediators occurred prior to changes in the relevant outcomes in a treatment where rapid mediational effects are likely. This enabled in‐depth, longitudinal examination of specific treatment procedures and the processes they were hypothesized to affect. Case‐by‐case extraction of data in order to deal with the challenge of treatment personalization was a further strength.

## 5 CONCLUSIONS

The findings add further support for the clinical importance of the “regular eating” procedure in reducing binge‐eating frequency in CBT‐E. While the data do not support the three other mediational hypotheses, they are not inconsistent with them. Investigating mediation within an RCT is potentially an efficient and cost‐effective way of further understanding how treatments work to help improve their potency. Such designs should be considered in future trials of CBT‐E and IPT‐ED. Studies should carefully consider what processes should be measured and when so that all key processes are assessed at the relevant time points to better examine the impact of multidimensional treatment procedures over time.

## CONFLICT OF INTEREST

The authors declare no potential conflict of interests.

## Supporting information


**Data S1**: Supporting Information.

## Data Availability

The authors elect to not share data due to privacy/ethical restrictions.
