# Supplementary material for: Mediators of change in cognitive behavior therapy and interpersonal psychotherapy for eating disorders: A secondary analysis of a transdiagnostic randomized controlled trial
Source: Int J Eat Disord. 2020 Nov 5;53(12):1928–40. doi: 10.1002/eat.23390 (PMC7756462; doi:10.1002/eat.23390)
Supplement: Supplementary file 1 — Data S1: Supporting Information. [file EAT-53-1928-s001.docx]

**SUPPORTING INFORMATION FOR:**

**Mediators of Change in Cognitive Behaviour Therapy and Interpersonal Psychotherapy for Eating Disorders: A Secondary Analysis of a Transdiagnostic Randomised Controlled Trial**

Katy Sivyer , Elizabeth Allen, Zafra Cooper, Suzanne Bailey-Straebler, Marianne E. O’Connor, Christopher G. Fairburn,^,^ Rebecca Murphy

This file includes summaries of the outputs for the statistical analyses reported in the main paper for [hypothesis 1](#_Statistical_Output_and), [hypothesis 2](#_Statistical_Output_and_1), [hypothesis 3](#_Statistical_Output_and_2) and [hypothesis 4](#_Statistical_Output_and_3). Quick links to specific outputs can be found in the **contents table below.**

**ABREVIATIONS**

CBT-E = Enhanced cognitive behaviour therapy

IPT-ED = Interpersonal psychotherapy for eating disorders

**OUTPUTS**

[Hypothesis 1: Regular Eating as a Mediator of the Effect of Enhanced Cognitive Behaviour Therapy on Frequency of Binge Eating 3](#_Toc47719871)

[Table 1: Multi-level model of the impact of the intervention (CBT-E) on the purported mediator (regular eating) 3](#_Toc47719872)

[Table 2: Multi-level model of the impact of the intervention (CBT-E) on the outcome (binge eating frequency) 4](#_Toc47719873)

[Table 3: Autoregressive structural equation model of the relationships between the intervention (CBT-E), the purported mediator (regular eating; RE) and the outcome (binge eating frequency; BE) 5](#_Toc47719874)

[Hypothesis 2: Weighing Frequency as a Mediator of the Effect of Enhanced Cognitive Behavioural Therapy on Concern about Weight 7](#_Toc47719875)

[Table 4: Multi-level model of the impact of the intervention (CBT-E) on the purported mediator (weighing frequency) 7](#_Toc47719876)

[Table 5: Multi-level model of the impact of the intervention (CBT-E) on the outcome (concern about weight) 8](#_Toc47719877)

[Table 6: Autoregressive structural equation model of the relationships between the intervention (CBT-E), the purported mediator (weighing frequency; FW) and the outcome (concern about weight; CW) 9](#_Toc47719878)

[Statistical Output and Stata Code for Hypothesis 3: Shape Checking as a Mediator of the Effect of Enhanced Cognitive Behavioural Therapy on Concern about Shape 13](#_Toc47719879)

[Table 7: Multi-level model of the impact of the shape checking intervention on the purported mediator (shape checking) 13](#_Toc47719880)

[Table 8: Multi-level model of the impact of the shape checking procedure on the outcome (concern about shape) 14](#_Toc47719881)

[Table 9: Autoregressive partial mediation model of the relationships between the shape checking intervention, shape checking at the previous week and within the same week, and concern about shape at time (T) using full information maximum likelihood estimation for missing data 15](#_Toc47719882)

[Hypothesis 4: Interpersonal Problem Severity as a Mediator of the Effect of Interpersonal Psychotherapy for Eating Disorders on Eating Disorder Psychopathology 19](#_Toc47719883)

[Table 10: Multi-level model of the impact of IPT-ED on the purported mediator (interpersonal problem severity) 19](#_Toc47719884)

[Table 11: Multi-level model of the impact of IPT-ED on the outcome (eating disorder psychopathology) 20](#_Toc47719885)

[Table 12: Autoregressive partial mediation model of the relationships IPT-ED, interpersonal problem severity at the previous time point and within the same time point, and eating disorder psychopathology using full information maximum likelihood estimation for missing data 21](#_Toc47719886)

# Hypothesis 1: Regular Eating as a Mediator of the Effect of Enhanced Cognitive Behaviour Therapy on Frequency of Binge Eating

## Table 1: Multi-level model of the impact of the intervention (CBT-E) on the purported mediator (regular eating)

| **Model Components** | **Variable Details** | **B** | **Boot. SE** | **p** | **Bias-corrected 95% CI of B** | |
| --- | --- | --- | --- | --- | --- | --- |
|  |  |  |  |  | Lower | Upper |
| Baseline predictors | Treatment allocation (CBT-E vs. IPT-ED) | 0.71 | 0.19 | .000 | 0.34 | 1.07 |
|  | Eating disorder diagnosis (BN vs. EDNOS) | -0.59 | 0.16 | .000 | -0.92 | -0.27 |
|  | Depression | -0.02 | 0.01 | .019 | -0.04 | -0.00 |
| Impact of treatment week | Treatment week (linear trend) | 0.19 | 0.13 | .150 | -0.06 | 0.45 |
|  | Treatment week (quadratic trend) | -0.06 | 0.04 | .197 | -0.14 | 0.02 |
| Impact of treatment allocation | CBT-E*treatment week (linear trend) | 1.12 | 0.21 | .000 | 0.68 | 1.51 |
|  | CBT-E*treatment week (quadratic trend) | -0.23 | 0.07 | .001 | -0.34 | -0.09 |
| Constant | Patients in IPT-ED with EDNOS average depression at baseline | 2.76 | 0.15 | .000 | 2.45 | 3.04 |
| Random effects  (log values) | SD (treatment week) | -1.78 | 0.11 | .000 | -1.91 | -1.91 |
|  | SD (constant) | 0.05 | 0.06 | .411 | -0.12 | 0.12 |
|  | Correlation (treatment week*constant) | -0.19 | 0.10 | .043 | -0.27 | 0.05 |
|  | SD (residual) | -0.50 | 0.06 | .000 | . | . |
| Statistics | AIC | 1175.14 | | | | |
|  | Number of patients | 117 | | | | |
|  | Number of observations | 439 | | | | |
|  | Number of bootstraps | 1000/1000 | | | | |

*Notes.* B=unstandardized coefficient, *p*-value based on 95% confidence interval, CI=confidence interval. BN=bulimia nervosa. EDNOS=eating disorder not otherwise specified.

## Table 2: Multi-level model of the impact of the intervention (CBT-E) on the outcome (binge eating frequency)

| **Model Components** | **Variable Details** | **B** | **Boot. SE** | **p** | **Bias-corrected 95% CI of B** | |
| --- | --- | --- | --- | --- | --- | --- |
|  |  |  |  |  | Lower | Upper |
| Baseline predictors | Treatment allocation (CBT-E vs. IPT-ED) | -0.43 | 0.43 | .316 | -1.31 | 0.41 |
|  | Weight concern | -0.41 | 0.13 | .002 | -0.66 | -0.14 |
|  | Eating disorder diagnosis (BN vs. EDNOS) | 2.43 | 0.49 | .000 | 1.51 | 3.44 |
|  | Presence of binge eating | 1.66 | 0.38 | .000 | 0.97 | 2.47 |
|  | Age | -0.06 | 0.02 | .009 | -0.12 | -0.02 |
| Impact of treatment week | Treatment week (linear trend) | 0.75 | 0.35 | .032 | 0.12 | 1.50 |
|  | Treatment week (quadratic trend) | -0.25 | 0.12 | .035 | -0.50 | -0.03 |
| Impact of baseline predictors during treatment | BN*treatment week (linear trend) | -0.34 | 0.17 | .048 | -0.71 | -0.02 |
|  | Presence of binge eating*treatment week (linear trend) | -0.20 | 0.10 | .041 | -0.40 | -0.01 |
|  | Age*treatment week (linear trend) | 0.02 | 0.01 | .002 | 0.01 | 0.03 |
| Impact of treatment allocation | CBT-E*treatment week (linear trend) | -1.40 | 0.51 | .007 | -2.47 | -0.47 |
|  | CBT-E*treatment week (quadratic trend) | 0.43 | 0.16 | .006 | 0.12 | 0.75 |
| Constant | Patients in IPT-ED with EDNOS, no binge eating, average weight concern and age at baseline | 0.56 | 0.34 | .105 | -0.19 | 1.16 |
| Random effects  (log values) | SD (treatment week) | -0.47 | 0.11 | .000 | -0.73 | -0.47 |
|  | SD (constant) | 0.88 | 0.08 | .000 | 0.61 | 0.97 |
|  | Correlation (treatment week*constant) | -0.63 | 0.13 | .000 | -0.86 | -0.32 |
|  | SD (residual) | 0.50 | 0.09 | .000 | 0.51 | 0.57 |
| Statistics | AIC | 2060.64 | | | | |
|  | Number of patients | 119 | | | | |
|  | Number of observations | 450 | | | | |
|  | Number of bootstraps | 1000/1000 | | | | |

*Notes.* B=unstandardized coefficient, *p*-value based on 95% confidence interval, CI=confidence interval. BN=bulimia nervosa. EDNOS=eating disorder not otherwise specified.

## Table 3: Autoregressive structural equation model of the relationships between the intervention (CBT-E), the purported mediator (regular eating; RE) and the outcome (binge eating frequency; BE)

| **Model Components** | **B** | **Boot.**  **SE** | **Bias-corrected 95% CI of B** | | **β** |
| --- | --- | --- | --- | --- | --- |
|  |  |  | Lower | Upper |  |
| RE1🡪RE2 | 0.64 | 0.08 | 0.48 | 0.79 | 0.57 |
| TX🡪RE2 | 1.21 | 0.18 | 0.86 | 1.58 | 0.41 |
| Depression🡪RE2 | 0.00 | 0.01 | -0.02 | 0.02 | 0.02 |
| ED diagnosis🡪RE2 | -0.29 | 0.17 | -0.67 | 0.03 | -0.10 |
| RE2🡪 BE2 | -0.71 | 0.28 | -1.26 | -0.22 | -0.33 |
| RE1🡪BE2 | 0.31 | 0.25 | -0.18 | 0.80 | 0.13 |
| BE1🡪BE2 | 0.52 | 0.10 | 0.32 | 0.71 | 0.55 |
| TX🡪 BE2 | -0.11 | 0.53 | -1.16 | 0.91 | -0.02 |
| ED diagnosis🡪 BE2 | 0.74 | 0.47 | -0.22 | 1.74 | 0.11 |
| BE Presence🡪 BE2 | 0.93 | 0.35 | 0.37 | 1.75 | 0.11 |
| WC🡪 BE2 | -0.10 | 0.18 | -0.48 | 0.23 | -0.04 |
| Age🡪BE2 | 0.00 | 0.02 | -0.04 | 0.05 | 0.00 |
| RE2🡪RE3 | 1.01 | 0.08 | 0.86 | 1.20 | 0.92 |
| TX🡪RE3 | 0.33 | 0.22 | -0.12 | 0.70 | 0.10 |
| Depression🡪RE3 | -0.01 | 0.01 | -0.02 | 0.01 | -0.03 |
| ED diagnosis🡪RE3 | -0.18 | 0.17 | -0.49 | 0.20 | -0.05 |
| RE2🡪 BE3 | 0.71 | 0.33 | 0.15 | 1.52 | 0.33 |
| BE2🡪BE3 | 0.78 | 0.25 | 0.38 | 1.35 | 0.78 |
| RE3🡪BE3 | -0.76 | 0.28 | -1.34 | -0.23 | -0.39 |
| TX🡪BE3 | -0.32 | 0.63 | -1.88 | 0.75 | -0.05 |
| ED diagnosis🡪BE3 | 0.07 | 0.66 | -1.20 | 1.38 | 0.01 |
| BE Presence🡪BE3 | -0.25 | 0.49 | -1.35 | 0.61 | -0.03 |
| WC 🡪BE3 | 0.03 | 0.19 | -0.38 | 0.37 | 0.01 |
| Age🡪BE3 | -0.01 | 0.02 | -0.05 | 0.03 | -0.03 |
| RE3🡪RE4 | 0.91 | 0.07 | 0.78 | 1.06 | 0.94 |
| TX🡪RE4 | 0.25 | 0.24 | -0.25 | 0.64 | 0.08 |
| Depression🡪RE4 | 0.01 | 0.01 | -0.01 | 0.02 | 0.06 |
| ED diagnosis🡪RE4 | 0.17 | 0.15 | -0.14 | 0.45 | 0.05 |
| RE3🡪 BE4 | 0.53 | 0.28 | -0.04 | 1.08 | 0.33 |
| BE3🡪BE4 | 0.74 | 0.15 | 0.50 | 1.10 | 0.91 |
| RE4🡪BE4 | -0.71 | 0.31 | -1.36 | -0.18 | -0.44 |
| TX🡪BE4 | 0.95 | 0.48 | 0.07 | 1.92 | 0.18 |
| ED diagnosis🡪BE4 | -0.20 | 0.39 | -0.96 | 0.62 | -0.04 |
| BE Presence🡪BE4 | 0.22 | 0.36 | -0.54 | 0.91 | 0.03 |
| WC 🡪BE4 | -0.28 | 0.18 | -0.69 | 0.03 | -0.15 |
| Age🡪BE4 | 0.02 | 0.02 | -0.01 | 0.07 | 0.07 |
| Var(e.RE2) | 0.79 | 0.09 | 0.67 | 1.04 | 0.36 |
| Var(e.BE2) | 4.19 | 0.64 | 3.25 | 6.05 | 0.41 |
| Var(e.RE3) | 0.62 | 0.11 | 0.46 | 0.92 | 0.23 |
| Var(e.BE3) | 4.43 | 1.32 | 2.63 | 7.71 | 0.44 |
| Var(e.RE4) | 0.52 | 0.10 | 0.36 | 0.74 | 0.21 |
| Var(e.BE4) | 3.49 | 1.06 | 1.96 | 7.60 | 0.52 |
| Cov(e.RE2*e.RE3) | -0.32 | 0.10 | -0.54 | -0.14 | -0.45 |
| Cov(e.BE2*e.BE3) | -0.82 | 0.93 | -3.22 | 0.49 | -0.19 |
| Cov(e.RE3*e.RE4) | -0.21 | 0.08 | -0.37 | -0.08 | -0.37 |
| Cov(e.BE3*e.BE4) | -1.73 | 0.87 | -4.16 | -0.46 | -0.44 |
| Cov(RE1*BE1) | -1.79 | 0.43 | -2.71 | -0.93 | -0.40 |
| Cov(RE1*TX) | 0.18 | 0.06 | 0.07 | 0.30 | 0.27 |
| Cov(RE1*Depression) | -3.72 | 1.31 | -6.37 | -1.27 | -0.26 |
| Cov(RE1*ED diagnosis) | -0.13 | 0.06 | -0.24 | -0.02 | -0.20 |
| Cov(RE1*BE Presence) | -0.04 | 0.05 | -0.12 | 0.07 | -0.07 |
| Cov(RE1*WC) | 0.14 | 0.18 | -0.17 | 0.57 | 0.08 |
| Cov(RE1*Age) | 0.10 | 1.02 | -2.09 | 1.93 | 0.01 |
| Cov(BE1*TX) | -0.12 | 0.15 | -0.44 | 0.14 | -0.07 |
| Cov(BE1*Depression) | 0.39 | 3.58 | -6.71 | 7.14 | 0.01 |
| Cov(BE1*ED diagnosis) | 0.71 | 0.14 | 0.44 | 1.02 | 0.43 |
| Cov(BE1*BE Presence) | 0.43 | 0.09 | 0.26 | 0.64 | 0.33 |
| Cov(BE1*WC) | -1.09 | 0.45 | -2.00 | -0.24 | -0.23 |
| Cov(BE1*Age) | -5.07 | 2.11 | -9.34 | -0.97 | -0.20 |
| Cov(TX*Depression) | -0.36 | 0.49 | -1.42 | 0.49 | -0.07 |
| Cov(TX*ED diagnosis) | -0.01 | 0.02 | -0.05 | 0.03 | -0.05 |
| Cov(TX*BE Presence) | 0.01 | 0.02 | -0.02 | 0.04 | 0.06 |
| Cov(TX*WC) | 0.07 | 0.06 | -0.05 | 0.20 | 0.11 |
| Cov(TX*Age) | -0.48 | 0.31 | -1.11 | 0.13 | -0.13 |
| Cov(Depression*ED diagnosis) | 0.59 | 0.46 | -0.34 | 1.45 | 0.11 |
| Cov(Depression*BE Presence) | 0.50 | 0.35 | -0.18 | 1.21 | 0.12 |
| Cov(Depression*WC) | 4.74 | 1.48 | 1.99 | 7.90 | 0.31 |
| Cov(Depression*Age) | 12.98 | 8.02 | -2.77 | 28.76 | 0.16 |
| Cov(ED diagnosis*BE Presence) | 0.08 | 0.01 | 0.06 | 0.11 | 0.40 |
| Cov(ED diagnosis*WC) | -0.02 | 0.06 | -0.15 | 0.08 | -0.03 |
| Cov(ED diagnosis*Age) | -0.37 | 0.32 | -0.98 | 0.25 | -0.10 |
| Cov(BE Presence*WC) | 0.01 | 0.05 | -0.08 | 0.09 | 0.01 |
| Cov(BE Presence*Age) | -0.27 | 0.30 | -0.93 | 0.26 | -0.09 |
| Cov(WC*Age) | 0.45 | 1.10 | -2.43 | 2.17 | 0.04 |
| Successful bootstraps | 892/1000 | | | | |

*Notes.* B=unstandardized coefficient. SE=standard error. CI=confidence interval. β=standardised coefficient. RE# = regular eating where # indicates intervention week. BE# = binge eating frequency where # indicates intervention week. TX=treatment allocation, BE Presence=Presence of binge eating in the month prior to starting treatment, WC = weight concern at baseline.

# **Hypothesis 2: Weighing Frequency as a Mediator of the Effect of Enhanced Cognitive Behavioural Therapy on Concern about Weight**

## Table 4: Multi-level model of the impact of the intervention (CBT-E) on the purported mediator (weighing frequency)

| **Model Components** | **Variable Details** | **B** | **Boot. SE** | **p** | **Bias-corrected 95% CI of B** | |
| --- | --- | --- | --- | --- | --- | --- |
|  |  |  |  |  | Lower | Upper |
| Baseline predictors | Treatment allocation (CBT-E vs. IPT-ED) | 0.68 | 0.70 | .328 | -0.67 | 1.91 |
|  | Frequent weigher status (frequent vs. non-frequent weigher) | 6.19 | 0.85 | .000 | 4.49 | 7.67 |
|  | Self-esteem (high vs. low self-esteem) | -1.80 | 0.76 | .017 | -3.25 | -0.31 |
| Impact of treatment week | Treatment week (linear trend) | 0.04 | 0.24 | .854 | -0.46 | 0.50 |
|  | Treatment week (quadratic) | 0.02 | 0.06 | .694 | -0.09 | 0.14 |
| Impact of baseline predictors during treatment | Weight concern (linear trend) | -0.28 | 0.12 | .017 | -0.53 | -0.08 |
|  | Weight concern (quadratic trend) | 0.06 | 0.03 | .012 | 0.02 | 0.12 |
| Moderators of treatment | Low self-esteem in CBT-E | -1.70 | 0.76 | .027 | -3.07 | -0.23 |
|  | Non-frequent weigher in CBT-E*treatment week (linear trend) | 0.48 | 0.28 | .094 | -0.05 | 1.06 |
|  | Non-frequent weigher in CBT-E*treatment week (quadratic trend) | -0.10 | 0.06 | .094 | -0.23 | 0.02 |
|  | Frequent weigher in IPT-ED*treatment week (linear trend) | -0.51 | 0.48 | .287 | -1.40 | 0.46 |
|  | Frequent weigher in IPT-ED*treatment week (quadratic trend) | -0.05 | 0.10 | .574 | -0.24 | 0.13 |
|  | Frequent weigher in CBT-E*treatment week (linear trend) | -3.25 | 0.91 | .000 | -5.00 | -1.56 |
|  | Frequent weigher in CBT-E*treatment week (quadratic trend) | 0.41 | 0.14 | .004 | 0.16 | 0.72 |
| Constant | Non-frequent weighers in IPT-ED with low self-esteem and average weight concern at baseline | 1.49 | 0.64 | .020 | 0.28 | 2.75 |
| Random effects  (log values) | SD (treatment week) | -0.16 | 0.29 | .582 | -1.03 | 0.08 |
|  | SD (constant) | 1.42 | 0.21 | .000 | 0.74 | 1.63 |
|  | Correlation (treatment week*constant) | -1.05 | 0.35 | .003 | -1.83 | -0.42 |
|  | SD (residual) | 0.54 | 0.14 | .000 | 0.39 | 0.75 |
| Statistics | AIC | 3115.80 | | | | |
|  | Number of patients | 114 | | | | |
|  | Number of observations | 658 | | | | |
|  | Number of bootstraps | 1000/1000 | | | | |

Notes. B=unstandardized coefficient, p-value based on 95% confidence interval, CI=confidence interval.

## Table 5: Multi-level model of the impact of the intervention (CBT-E) on the outcome (concern about weight)

| **Model Components** | **Variable Details** | **B** | **Boot. SE** | **p** | **Bias-corrected 95% CI of B** | |
| --- | --- | --- | --- | --- | --- | --- |
|  |  |  |  |  | Lower | Upper |
| Baseline predictors | Treatment allocation (CBT-E vs. IPT-ED) | 0.00 | 0.19 | .980 | -0.39 | 0.37 |
|  | Weight concern | 0.68 | 0.06 | .000 | 0.55 | 0.80 |
|  | Weight concern (quadratic) | -0.10 | 0.03 | .001 | -0.16 | -0.03 |
|  | Self-esteem | -0.02 | 0.03 | .385 | -0.07 | 0.03 |
|  | Self-esteem (quadratic) | 0.02 | 0.01 | .011 | 0.00 | 0.03 |
| Impact of treatment week | Treatment week (linear trend) | -0.36 | 0.07 | .000 | -0.52 | -0.22 |
|  | Treatment week (quadratic) | 0.03 | 0.01 | .015 | 0.01 | 0.06 |
| Impact of treatment allocation | CBT-E*treatment week (linear trend) | 0.04 | 0.05 | .434 | -0.06 | 0.13 |
| Constant | Patients in IPT-ED with average weight concern and self-esteem at baseline | 4.61 | 0.18 | .000 | 4.27 | 4.96 |
| Random effects  (log values) | SD (treatment week) | -1.27 | 0.09 | .000 | -1.53 | -1.19 |
|  | SD (constant) | 0.12 | 0.08 | .146 | -0.16 | 0.23 |
|  | Correlation (treatment week*constant) | -0.26 | 0.10 | .011 | -0.37 | 0.09 |
|  | SD (residual) | -0.13 | 0.05 | .009 | -0.13 | -0.08 |
| Statistics | AIC | 2321.72 | | | | |
|  | Number of patients | 125 | | | | |
|  | Number of observations | 718 | | | | |
|  | Number of bootstraps | 1000/1000 | | | | |

*Notes.* B=unstandardized coefficient, *p*-value based on 95% confidence interval, CI=confidence interval.

## Table 6: Autoregressive structural equation model of the relationships between the intervention (CBT-E), the purported mediator (weighing frequency; FW) and the outcome (concern about weight; CW)

| **Model Components** | **B** | **Boot.**  **SE** | **Bias-corrected 95% CI of B** | | **β** |
| --- | --- | --- | --- | --- | --- |
|  |  |  | Lower | Upper |  |
| FW1🡪FW2 | 0.69 | 0.16 | 0.32 | 1.01 | 0.81 |
| TX🡪FW2 | 0.61 | 0.73 | -0.79 | 2.08 | 0.07 |
| Freq weigher🡪FW2 | 1.48 | 0.95 | -0.26 | 3.61 | 0.16 |
| RSE group🡪FW2 | -0.84 | 0.70 | -2.26 | 0.57 | -0.09 |
| Freq weigher*TX🡪FW2 | -3.73 | 1.14 | -6.32 | -1.65 | -0.32 |
| RSE group*TX🡪FW2 | 0.34 | 1.01 | -1.78 | 2.21 | 0.03 |
| FW2🡪CW2 | 0.04 | 0.04 | -0.04 | 0.12 | 0.08 |
| FW1🡪CW2 | -0.00 | 0.04 | -0.09 | 0.07 | -0.01 |
| WW1🡪CW2 | 0.70 | 0.11 | 0.47 | 0.91 | 0.60 |
| TX🡪CW2 | 0.08 | 0.20 | -0.34 | 0.48 | 0.02 |
| WC🡪CW2 | 0.27 | 0.14 | 0.01 | 0.55 | 0.20 |
| RSE🡪CW2 | -0.01 | 0.03 | -0.06 | 0.05 | -0.01 |
| WC(quad)🡪CW2 | -0.05 | 0.05 | -0.15 | 0.05 | -0.07 |
| RSE(quad)🡪CW2 | 0.01 | 0.01 | 0.00 | 0.02 | 0.14 |
| FW2🡪FW3 | 0.37 | 0.20 | 0.09 | 0.72 | 0.55 |
| TX🡪FW3 | -0.35 | 0.53 | -1.72 | 0.52 | -0.06 |
| Freq Weigher🡪FW3 | 1.92 | 1.15 | -0.22 | 3.94 | 0.30 |
| RSE group🡪FW3 | -0.09 | 0.61 | -1.65 | 0.82 | -0.02 |
| Freq weigher*TX🡪FW3 | -2.36 | 1.14 | -4.53 | -0.21 | -0.31 |
| RSE group*TX🡪FW3 | 0.76 | 0.71 | -0.32 | 2.57 | 0.11 |
| FW2🡪CW3 | -0.06 | 0.06 | -0.14 | 0.08 | -0.16 |
| WW2🡪CW3 | 0.97 | 0.14 | 0.68 | 1.25 | 0.98 |
| FW3🡪CW3 | 0.06 | 0.08 | -0.09 | 0.20 | 0.10 |
| TX🡪CW3 | 0.01 | 0.25 | -0.48 | 0.47 | 0.00 |
| WC🡪CW3 | -0.08 | 0.13 | -0.37 | 0.15 | -0.06 |
| RSE🡪CW3 | 0.03 | 0.04 | -0.04 | 0.10 | 0.06 |
| WC(quad)🡪CW3 | 0.01 | 0.05 | -0.08 | 0.11 | 0.01 |
| RSE(quad)🡪CW3 | -0.01 | 0.01 | -0.03 | 0.00 | -0.14 |
| FW3🡪FW4 | 1.22 | 0.23 | 0.76 | 1.72 | 1.04 |
| TX🡪FW4 | -0.79 | 0.46 | -1.99 | -0.08 | -0.11 |
| Freq Weigher🡪FW4 | -0.87 | 0.84 | -2.94 | 0.55 | -0.12 |
| RSE group🡪FW4 | -1.38 | 0.60 | -2.86 | -0.38 | -0.19 |
| Freq weigher*TX🡪FW4 | 0.72 | 0.84 | -0.71 | 2.75 | 0.08 |
| RSE group*TX🡪FW4 | 1.26 | 0.63 | 0.15 | 2.72 | 0.16 |
| FW3🡪CW4 | -0.15 | 0.06 | -0.28 | -0.03 | -0.24 |
| WW3🡪CW4 | 0.92 | 0.12 | 0.70 | 1.17 | 0.93 |
| FW3🡪CW4 | 0.16 | 0.05 | 0.07 | 0.26 | 0.31 |
| TX🡪CW4 | 0.29 | 0.23 | -0.16 | 0.72 | 0.08 |
| WC🡪CW4 | 0.09 | 0.10 | -0.12 | 0.28 | 0.07 |
| RSE🡪CW4 | -0.00 | 0.03 | -0.06 | 0.06 | -0.01 |
| WC(quad)🡪CW4 | 0.04 | 0.05 | -0.04 | 0.13 | 0.07 |
| RSE(quad)🡪CW4 | 0.00 | 0.01 | -0.01 | 0.02 | 0.05 |
| FW4🡪FW5 | 0.87 | 0.10 | 0.67 | 1.02 | 0.91 |
| TX🡪FW5 | -0.56 | 0.79 | -2.58 | 0.50 | -0.08 |
| Freq Weigher🡪FW5 | -0.30 | 0.68 | -1.83 | 0.83 | -0.04 |
| RSE group🡪FW5 | -0.18 | 0.66 | -1.75 | 0.84 | -0.03 |
| Freq weigher*TX🡪FW5 | 0.36 | 0.69 | -0.83 | 1.95 | 0.04 |
| RSE group*TX🡪FW5 | 0.31 | 0.67 | -0.75 | 1.96 | 0.04 |
| FW4🡪CW5 | -0.05 | 0.07 | -0.21 | 0.05 | -0.09 |
| WW4🡪CW5 | 0.70 | 0.16 | 0.38 | 0.99 | 0.68 |
| FW5🡪CW5 | 0.07 | 0.07 | -0.07 | 0.23 | 0.13 |
| TX🡪CW5 | -0.19 | 0.25 | -0.71 | 0.27 | -0.05 |
| WC🡪CW5 | 0.17 | 0.15 | -0.13 | 0.49 | 0.12 |
| RSE🡪CW5 | -0.01 | 0.04 | -0.08 | 0.06 | -0.02 |
| WC(quad)🡪CW5 | -0.09 | 0.05 | -0.19 | 0.00 | -0.13 |
| RSE(quad)🡪CW5 | 0.01 | 0.01 | 0.00 | 0.02 | 0.11 |
| FW5🡪FW6 | 0.70 | 0.14 | 0.42 | 1.02 | 0.87 |
| TX🡪FW6 | -0.08 | 0.40 | -0.95 | 0.60 | -0.01 |
| Freq Weigher🡪FW6 | 0.10 | 0.46 | -0.77 | 1.04 | 0.02 |
| RSE group🡪FW6 | -0.50 | 0.41 | -1.33 | 0.25 | -0.09 |
| Freq weigher*TX🡪FW6 | -0.13 | 0.45 | -1.06 | 0.74 | -0.02 |
| RSE group*TX🡪FW6 | 0.38 | 0.43 | -0.39 | 1.21 | 0.06 |
| FW5🡪CW6 | -0.06 | 0.09 | -0.26 | 0.12 | -0.10 |
| WW5🡪CW6 | 1.21 | 0.19 | 0.92 | 1.71 | 1.15 |
| FW6🡪CW6 | 0.11 | 0.10 | -0.09 | 0.31 | 0.15 |
| TX🡪CW6 | 0.28 | 0.29 | -0.22 | 0.91 | 0.07 |
| WC🡪CW6 | -0.13 | 0.16 | -0.50 | 0.15 | -0.09 |
| RSE🡪CW6 | -0.00 | 0.05 | -0.10 | 0.08 | -0.01 |
| WC(quad)🡪CW6 | 0.11 | 0.07 | -0.02 | 0.26 | 0.15 |
| RSE(quad)🡪CW6 | -0.01 | 0.01 | -0.03 | 0.01 | -0.09 |
| Var(e.FW2) | 6.64 | 1.74 | 4.05 | 11.47 | 0.31 |
| Var(e.CW2) | 1.26 | 0.24 | 0.89 | 1.94 | 0.34 |
| Var(e.FW3) | 3.40 | 1.08 | 1.71 | 6.35 | 0.36 |
| Var(e.CW3) | 1.31 | 0.21 | 1.02 | 2.04 | 0.37 |
| Var(e.FW4) | 2.99 | 1.03 | 1.40 | 5.57 | 0.23 |
| Var(e.CW4) | 1.22 | 0.22 | 0.91 | 2.07 | 0.35 |
| Var(e.FW5) | 2.91 | 1.80 | 0.63 | 7.92 | 0.25 |
| Var(e.CW5) | 1.48 | 0.21 | 1.15 | 1.99 | 0.40 |
| Var(e.FW6) | 0.87 | 0.55 | 0.47 | 1.96 | 0.11 |
| Var(e.CW6) | 1.67 | 0.53 | 1.06 | 3.57 | 0.41 |
| Cov(e.FW2*e.FW3) | 1.89 | 1.17 | -0.13 | 4.10 | 0.40 |
| Cov(e.CW2*e.CW3) | -0.38 | 0.19 | -0.86 | -0.08 | -0.29 |
| Cov(e.FW3*e.FW4) | -1.47 | 0.57 | -2.68 | -0.50 | -0.46 |
| Cov(e.CW3*e.CW4) | -0.54 | 0.23 | -1.20 | -0.21 | -0.43 |
| Cov(e.FW4*e.FW5) | -0.53 | 0.35 | -1.31 | 0.08 | -0.18 |
| Cov(e.CW4*e.CW5) | -0.21 | 0.19 | -0.64 | 0.10 | -0.15 |
| Cov(e.FW5*e.FW6) | 0.57 | 0.76 | -0.24 | 2.68 | 0.36 |
| Cov(e.CW5*e.CW6) | -0.90 | 0.39 | -2.00 | -0.34 | -0.57 |
| Cov(FW1*WW1) | 1.95 | 0.62 | 0.96 | 3.67 | 0.22 |
| Cov(FW1*TX) | -0.10 | 0.25 | -0.52 | 0.47 | -0.04 |
| Cov(FW1*Freq Weigher) | 1.44 | 0.23 | 1.07 | 2.01 | 0.55 |
| Cov(FW1*RSE group) | -0.43 | 0.26 | -1.01 | 0.03 | -0.16 |
| Cov(FW1*Freq Weigher*TX) | 0.69 | 0.30 | 0.23 | 1.45 | 0.32 |
| Cov(FW1*RSE group*TX) | -0.35 | 0.19 | -0.74 | 0.02 | -0.14 |
| Cov(FW1*WC) | 2.00 | 0.78 | 0.83 | 4.18 | 0.26 |
| Cov(FW1*self-esteem) | -3.19 | 1.29 | -6.08 | -1.00 | -0.16 |
| Cov(FW1*WC(quad)) | -0.70 | 1.11 | -2.61 | 1.76 | -0.05 |
| Cov(FW1*self-esteem(quad)) | -9.56 | 5.82 | -23.97 | 0.20 | -0.09 |
| Cov(CW1*TX) | 0.05 | 0.07 | -0.08 | 0.20 | 0.06 |
| Cov(WW1*Freq Weigher) | 0.15 | 0.07 | 0.03 | 0.30 | 0.19 |
| Cov(CW1*RSE group) | -0.09 | 0.07 | -0.23 | 0.04 | -0.11 |
| Cov(CW1*Freq Weigher*TX) | 0.07 | 0.06 | -0.04 | 0.18 | 0.11 |
| Cov(CW1*RSE group*TX) | -0.01 | 0.06 | -0.14 | 0.12 | -0.01 |
| Cov(CW1*WC) | 1.53 | 0.27 | 1.01 | 2.06 | 0.67 |
| Cov(CW1*self-esteem) | -0.76 | 0.46 | -1.66 | 0.07 | -0.13 |
| Cov(CW1*WC(quad)) | -2.20 | 0.66 | -3.72 | -1.03 | -0.48 |
| Cov(CW1*self-esteem(quad)) | 0.39 | 2.12 | -3.84 | 4.66 | 0.01 |
| Cov(TX*Freq Weigher) | -0.01 | 0.02 | -0.05 | 0.03 | -0.05 |
| Cov(TX*RSE group) | 0.03 | 0.02 | -0.02 | 0.07 | 0.11 |
| Cov(TX*Freq Weigher*TX) | 0.10 | 0.02 | 0.07 | 0.13 | 0.51 |
| Cov(TX*RSE group*TX) | 0.16 | 0.02 | 0.13 | 0.19 | 0.68 |
| Cov(TX*WC) | 0.07 | 0.06 | -0.04 | 0.20 | 0.11 |
| Cov(TX*self-esteem) | 0.23 | 0.16 | -0.08 | 0.53 | 0.13 |
| Cov(TX*WC(quad)) | -0.13 | 0.12 | -0.39 | 0.10 | -0.09 |
| Cov(TX*self-esteem(quad)) | 0.04 | 0.86 | -1.83 | 1.55 | 0.00 |
| Cov(Freq Weigher*RSE group) | -0.04 | 0.02 | -0.08 | 0.01 | -0.15 |
| Cov(Freq Weigher*Freq Weigher*TX) | 0.11 | 0.02 | 0.08 | 0.14 | 0.58 |
| Cov(Freq Weighe *RSE group*TX) | -0.03 | 0.02 | -0.07 | 0.01 | -0.13 |
| Cov(Freq Weigher *WC) | 0.12 | 0.06 | 0.01 | 0.24 | 0.18 |
| Cov(Freq Weigher*self-esteem) | -0.26 | 0.16 | -0.57 | 0.04 | -0.15 |
| Cov(Freq Weigher*WC(quad)) | -0.29 | 0.11 | -0.53 | -0.11 | -0.21 |
| Cov(Freq Weigher *self-esteem(quad)) | -0.11 | 0.89 | -1.68 | 1.84 | -0.01 |
| Cov(RSE group*Freq Weigher*TX) | -0.01 | 0.02 | -0.05 | 0.02 | -0.07 |
| Cov(RSE group*RSE group*TX) | 0.13 | 0.01 | 0.11 | 0.16 | 0.58 |
| Cov(RSE group*WC) | -0.07 | 0.06 | -0.20 | 0.04 | -0.10 |
| Cov(RSE group*self-esteem) | 1.37 | 0.10 | 1.20 | 1.56 | 0.77 |
| Cov(RSE group*WC(quad)) | 0.06 | 0.12 | -0.17 | 0.32 | 0.04 |
| Cov(RSE group*self-esteem(quad)) | 0.27 | 0.75 | -1.11 | 1.75 | 0.03 |
| Cov(Freq Weigher*TX*RSE group*TX) | 0.04 | 0.02 | 0.01 | 0.08 | 0.22 |
| Cov(Freq Weigher*TX*WC) | 0.07 | 0.04 | -0.01 | 0.15 | 0.12 |
| Cov(Freq Weigher*TX*self-esteem) | -0.04 | 0.13 | -0.27 | 0.24 | -0.03 |
| Cov(Freq Weigher*TX*WC(quad)) | -0.15 | 0.07 | -0.30 | -0.01 | -0.13 |
| Cov(Freq Weigher*TX*self-esteem(quad)) | -0.03 | 0.78 | -1.24 | 1.93 | -0.00 |
| Cov(RSE group*TX*WC) | -0.02 | 0.06 | -0.15 | 0.08 | -0.03 |
| Cov(RSE group*TX*self-esteem) | 0.79 | 0.13 | 0.56 | 1.07 | 0.48 |
| Cov(RSE group*TX*WC(quad)) | -0.09 | 0.11 | -0.29 | 0.12 | -0.07 |
| Cov(RSE group*TX *self-esteem(quad)) | 0.36 | 0.86 | -1.20 | 2.26 | 0.04 |
| Cov(WC*self-esteem) | -0.90 | 0.51 | -2.02 | -0.05 | -0.18 |
| Cov(WC*WC(quad)) | -2.27 | 0.67 | -3.77 | -1.10 | -0.57 |
| Cov(WC*self-esteem(quad)) | -3.67 | 3.31 | -11.29 | 1.22 | -0.13 |
| Cov(self-esteem*WC(quad)) | 1.71 | 1.13 | -0.42 | 3.97 | 0.17 |
| Cov(self-esteem*self-esteem(quad)) | 31.43 | 14.67 | 6.10 | 63.19 | 0.45 |
| Cov(WC(quad)*self-esteem(quad)) | 6.81 | 7.99 | -5.05 | 26.18 | 0.13 |
| Successful bootstraps | 991/1000 | | | | |

*Notes.* B=unstandardized coefficient. SE=standard error. β=standardised coefficient. CI=confidence interval. FW# = weighing frequency where # indicates intervention week. CW# = concern about weight where # indicates intervention week. Freq weigher=frequent weigher status (frequent weigher vs. non-frequent weigher), RSE group=self-esteem group (high vs. low self-esteem), TX=treatment allocation, WC = weight concern at baseline, RSE=self-esteem at baseline, (quad)=quadratic trend in the covariate.

# Statistical Output and Stata Code for **Hypothesis 3: Shape Checking as a Mediator of the Effect of Enhanced Cognitive Behavioural Therapy on Concern about Shape**

## Table 7: Multi-level model of the impact of the shape checking intervention on the purported mediator (shape checking)

| **Model Components** | **Variable Details** | **B** | **Boot. SE** | **p** | **Bias-corrected 95% CI of B** | |
| --- | --- | --- | --- | --- | --- | --- |
|  |  |  |  |  | Lower | Upper |
| Baseline Predictors | Weight concern | 0.15 | 0.05 | .001 | 0.06 | 0.23 |
|  | Self-esteem | -0.05 | 0.02 | .000 | -0.08 | -0.02 |
|  | Eating disorder duration | -0.02 | 0.01 | .007 | -0.03 | -0.01 |
|  | Treatment allocation (CBT-E vs. IPT-ED) | 0.15 | 0.17 | .373 | -0.17 | 0.49 |
| Impact of treatment week | Treatment week (linear trend) | 0.01 | 0.02 | .767 | -0.03 | 0.05 |
|  | Treatment week (quadratic) | 0.00 | 0.00 | .267 | 0.00 | 0.00 |
| Impact of treatment allocation | CBT-E condition*Treatment week (linear trend) | -0.05 | 0.03 | .122 | -0.11 | 0.01 |
|  | CBT-E condition*Treatment week (quadratic trend) | 0.01 | 0.00 | .023 | 0.00 | 0.01 |
| Impact of shape checking procedure | Shape checking intervention week (linear trend) | -0.18 | 0.05 | .000 | -0.26 | -0.08 |
| Impact of baseline predictors during treatment/intervention | Self-esteem*Intervention week (linear trend) | 0.01 | 0.00 | .018 | 0.00 | 0.01 |
| Constant | Patients in IPT-ED with average weight concern and eating disorder duration at baseline | 3.11 | 0.11 | .000 | 2.87 | 3.32 |
| Random effects  (log values) | SD (treatment week) | -2.38 | 0.08 | .000 | -2.69 | -2.32 |
|  | SD (time) | -1.95 | 0.12 | .000 | -2.31 | -1.82 |
|  | SD (constant) | 0.02 | 0.05 | .638 | -0.13 | 0.09 |
|  | Correlation (treatment week*intervention week) | -0.79 | 0.11 | .000 | -0.90 | -0.48 |
|  | Correlation (treatment week*constant) | -0.53 | 0.08 | .000 | -0.65 | -0.31 |
|  | Correlation (intervention week*constant) | -0.12 | 0.11 | .267 | -0.56 | 0.00 |
|  | SD (residual) | -0.51 | 0.04 | .000 | -0.54 | -0.44 |
| Statistics | AIC | 4154.31 | | | | |
|  | Number of patients | 125 | | | | |
|  | Number of observations | 1849 | | | | |
|  | Number of bootstraps | 1000/1000 | | | | |

*Notes.* B=unstandardized coefficient, *p*-value based on 95% confidence interval, CI=confidence interval.

## Table 8: Multi-level model of the impact of the shape checking procedure on the outcome (concern about shape)

| **Model Components** | **Variable Details** | **B** | **Boot. SE** | **p** | **Bias-corrected 95% CI of B** | |
| --- | --- | --- | --- | --- | --- | --- |
|  |  |  |  |  | Lower | Upper |
| Baseline Predictors | Weight concern | 0.55 | 0.07 | .000 | 0.38 | 0.68 |
|  | Weight concern (quadratic trend) | -0.09 | 0.03 | .008 | -0.15 | -0.03 |
|  | Age | 0.02 | 0.01 | .005 | 0.01 | 0.04 |
|  | Depression | 0.01 | 0.01 | .100 | 0.00 | 0.02 |
|  | Importance of shape | -0.20 | 0.07 | .005 | -0.33 | -0.05 |
| Impact of treatment week | Treatment week (linear trend) | -0.13 | 0.02 | .000 | -0.17 | -0.08 |
|  | Treatment week (quadratic) | 0.00 | 0.00 | .007 | 0.00 | 0.01 |
| Impact of baseline predictors during treatment | Weight concern*Treatment week (linear trend) | -0.02 | 0.01 | .027 | -0.03 | 0.00 |
|  | Depression*Shape checking intervention week (linear trend) | -0.01 | 0.00 | .022 | -0.01 | 0.00 |
|  | Importance of shape*Shape checking intervention week (linear trend) | 0.04 | 0.02 | .008 | 0.02 | 0.08 |
| Impact of shape checking procedure | Shape checking intervention week (linear trend) | -0.09 | 0.03 | .002 | -0.14 | -0.03 |
| Constant | Patients in the control group with average weight concern, age, depression and importance of shape at baseline | 5.25 | 0.10 | .000 | 5.06 | 5.44 |
| Random effects  (log values) | SD (treatment week) | -2.06 | 0.11 | .000 | -2.31 | -2.00 |
|  | SD (time) | -1.56 | 0.11 | .000 | -1.94 | -1.47 |
|  | SD (constant) | -0.12 | 0.10 | .247 | -0.49 | 0.02 |
|  | Correlation (treatment week*intervention week) | -0.91 | 0.10 | .000 | -0.99 | -0.65 |
|  | Correlation (treatment week*constant) | -0.01 | 0.13 | .934 | -0.11 | 0.33 |
|  | Correlation (intervention week*constant) | -0.20 | 0.13 | .105 | -0.52 | -0.10 |
|  | SD (residual) | -0.06 | 0.04 | .151 | -0.09 | 0.00 |
| Statistics | AIC | 5751.51 | | | | |
|  | Number of patients | 127 | | | | |
|  | Number of observations | 1854 | | | | |
|  | Number of bootstraps | 1000/1000 | | | | |

*Notes.* B=unstandardized coefficient, *p*-value based on 95% confidence interval, CI=confidence interval.

## Table 9: Autoregressive partial mediation model of the relationships between the shape checking intervention, shape checking at the previous week and within the same week, and concern about shape at time (T) using full information maximum likelihood estimation for missing data

| **Model Components** | **B** | **Boot.**  **SE** | **Bias-corrected 95% CI of B** | | **β** |
| --- | --- | --- | --- | --- | --- |
|  |  |  | Lower | Upper |  |
| FC0🡪FC1 | 0.64 | 0.08 | 0.49 | 0.80 | 0.66 |
| SCINT🡪FC1 | 0.40 | 0.13 | 0.14 | 0.65 | 0.17 |
| WC🡪FC1 | 0.00 | 0.05 | -0.10 | 0.10 | -0.01 |
| EDDUR🡪FC1 | 0.00 | 0.01 | -0.02 | 0.01 | -0.01 |
| SELF-ESTEEM🡪FC1 | 0.00 | 0.02 | -0.04 | 0.04 | -0.01 |
| FCBASELINE🡪FC1 | 0.21 | 0.09 | 0.03 | 0.38 | 0.23 |
| FC1🡪CS1 | 0.34 | 0.15 | 0.09 | 0.69 | 0.22 |
| FC0🡪CS1 | -0.05 | 0.15 | -0.37 | 0.22 | -0.04 |
| CS0🡪CS1 | 0.73 | 0.08 | 0.56 | 0.85 | 0.70 |
| SCINT🡪CS1 | -0.01 | 0.24 | -0.50 | 0.43 | 0.00 |
| WC🡪CS1 | -0.13 | 0.12 | -0.38 | 0.08 | -0.10 |
| WC(QUAD)🡪CS1 | -0.02 | 0.05 | -0.12 | 0.07 | -0.04 |
| AGE🡪CS1 | -0.01 | 0.01 | -0.04 | 0.01 | -0.03 |
| DEP🡪CS1 | -0.01 | 0.01 | -0.03 | 0.01 | -0.06 |
| IMP SHAPE🡪CS1 | 0.15 | 0.08 | -0.01 | 0.31 | 0.13 |
| FCBASELINE🡪CS1 | 0.12 | 0.10 | -0.07 | 0.31 | 0.08 |
| FC1🡪FC2 | 0.93 | 0.12 | 0.71 | 1.21 | 0.98 |
| SCINT🡪FC2 | -0.17 | 0.17 | -0.53 | 0.16 | -0.08 |
| WC🡪FC2 | 0.01 | 0.06 | -0.09 | 0.11 | 0.01 |
| EDDUR🡪FC2 | 0.00 | 0.01 | -0.02 | 0.01 | -0.02 |
| SELF-ESTEEM🡪FC2 | 0.00 | 0.02 | -0.04 | 0.04 | -0.01 |
| FCBASELINE🡪FC2 | -0.19 | 0.11 | -0.42 | 0.01 | -0.21 |
| FC1🡪CS2 | -0.06 | 0.15 | -0.38 | 0.21 | -0.04 |
| CS1🡪CS2 | 0.79 | 0.12 | 0.57 | 1.06 | 0.78 |
| FC2🡪CS2 | 0.35 | 0.14 | 0.06 | 0.60 | 0.21 |
| SCINT🡪CS2 | 0.32 | 0.25 | -0.17 | 0.84 | 0.09 |
| WC🡪CS2 | 0.14 | 0.14 | -0.16 | 0.39 | 0.11 |
| WC(QUAD)🡪CS2 | 0.03 | 0.06 | -0.08 | 0.14 | 0.04 |
| AGE🡪CS2 | 0.01 | 0.01 | -0.01 | 0.04 | 0.05 |
| DEP🡪CS2 | -0.02 | 0.01 | -0.04 | 0.00 | -0.14 |
| IMP SHAPE🡪CS2 | 0.00 | 0.12 | -0.25 | 0.24 | 0.00 |
| FCBASELINE🡪CS2 | -0.03 | 0.12 | -0.28 | 0.21 | -0.02 |
| FC2🡪FC3 | 0.85 | 0.11 | 0.62 | 1.05 | 0.83 |
| SCINT🡪FC3 | -0.49 | 0.16 | -0.82 | -0.18 | -0.22 |
| WC🡪FC3 | 0.05 | 0.06 | -0.05 | 0.17 | 0.07 |
| EDDUR🡪FC3 | 0.00 | 0.01 | -0.02 | 0.02 | 0.02 |
| SELF-ESTEEM🡪FC3 | 0.02 | 0.03 | -0.03 | 0.08 | 0.07 |
| FCBASELINE🡪FC3 | 0.09 | 0.07 | -0.04 | 0.25 | 0.10 |
| FC2🡪CS3 | -0.46 | 0.17 | -0.81 | -0.15 | -0.28 |
| CS2🡪CS3 | 0.98 | 0.19 | 0.62 | 1.39 | 0.98 |
| FC3🡪CS3 | 0.37 | 0.15 | 0.06 | 0.66 | 0.23 |
| SCINT🡪CS3 | -0.15 | 0.30 | -0.76 | 0.47 | -0.04 |
| WC🡪CS3 | 0.15 | 0.20 | -0.24 | 0.55 | 0.12 |
| WC(QUAD)🡪CS3 | 0.00 | 0.06 | -0.12 | 0.12 | 0.00 |
| AGE🡪CS3 | 0.01 | 0.02 | -0.03 | 0.04 | 0.02 |
| DEP🡪CS3 | 0.01 | 0.02 | -0.01 | 0.04 | 0.08 |
| IMP SHAPE🡪CS3 | -0.12 | 0.16 | -0.43 | 0.19 | -0.11 |
| FCBASELINE🡪CS3 | 0.03 | 0.22 | -0.39 | 0.45 | 0.02 |
| FC3🡪FC4 | 0.91 | 0.13 | 0.67 | 1.19 | 0.90 |
| SCINT🡪FC4 | -0.24 | 0.18 | -0.59 | 0.13 | -0.10 |
| WC🡪FC4 | 0.03 | 0.05 | -0.08 | 0.14 | 0.03 |
| EDDUR🡪FC4 | 0.01 | 0.01 | -0.01 | 0.03 | 0.08 |
| SELF-ESTEEM🡪FC4 | 0.02 | 0.02 | -0.03 | 0.06 | 0.06 |
| FCBASELINE🡪FC4 | 0.03 | 0.10 | -0.19 | 0.22 | 0.03 |
| FC3🡪CS4 | -0.78 | 0.21 | -1.21 | -0.41 | -0.48 |
| CS3🡪CS4 | 1.15 | 0.15 | 0.90 | 1.47 | 1.13 |
| FC4🡪CS4 | 0.64 | 0.17 | 0.31 | 0.97 | 0.39 |
| SCINT🡪CS4 | -0.32 | 0.30 | -0.95 | 0.28 | -0.09 |
| WC🡪CS4 | -0.33 | 0.21 | -0.77 | 0.06 | -0.25 |
| WC(QUAD)🡪CS4 | -0.01 | 0.07 | -0.13 | 0.13 | -0.01 |
| AGE🡪CS4 | -0.03 | 0.02 | -0.07 | 0.01 | -0.11 |
| DEP🡪CS4 | 0.03 | 0.02 | -0.01 | 0.06 | 0.16 |
| IMP SHAPE🡪CS4 | 0.22 | 0.16 | -0.07 | 0.56 | 0.20 |
| FCBASELINE🡪CS4 | -0.13 | 0.16 | -0.48 | 0.16 | -0.09 |
| FC4🡪FC5 | 0.93 | 0.15 | 0.62 | 1.19 | 0.92 |
| SCINT🡪FC5 | 0.05 | 0.21 | -0.35 | 0.49 | 0.02 |
| WC🡪FC5 | 0.05 | 0.07 | -0.09 | 0.16 | 0.06 |
| EDDUR🡪FC5 | -0.01 | 0.01 | -0.03 | 0.02 | -0.04 |
| SELF-ESTEEM🡪FC5 | 0.00 | 0.02 | -0.05 | 0.05 | 0.01 |
| FCBASELINE🡪FC5 | -0.01 | 0.10 | -0.22 | 0.17 | -0.01 |
| FC4🡪CS5 | -0.39 | 0.20 | -0.92 | -0.06 | -0.24 |
| CS4🡪CS5 | 0.87 | 0.12 | 0.68 | 1.15 | 0.86 |
| FC5🡪CS5 | 0.50 | 0.20 | 0.12 | 0.91 | 0.31 |
| SCINT🡪CS5 | -0.02 | 0.29 | -0.60 | 0.49 | -0.01 |
| WC🡪CS5 | 0.02 | 0.16 | -0.29 | 0.33 | 0.01 |
| WC(QUAD)🡪CS5 | -0.02 | 0.06 | -0.15 | 0.10 | -0.04 |
| AGE🡪CS5 | 0.04 | 0.02 | 0.01 | 0.09 | 0.17 |
| DEP🡪CS5 | -0.02 | 0.02 | -0.05 | 0.01 | -0.12 |
| IMP SHAPE🡪CS5 | -0.15 | 0.12 | -0.41 | 0.07 | -0.13 |
| FCBASELINE🡪CS5 | 0.15 | 0.12 | -0.07 | 0.37 | 0.10 |
| FC5🡪FC6 | 0.93 | 0.12 | 0.74 | 1.22 | 0.97 |
| SCINT🡪FC6 | -0.45 | 0.17 | -0.79 | -0.12 | -0.20 |
| WC🡪FC6 | 0.00 | 0.06 | -0.13 | 0.12 | 0.00 |
| EDDUR🡪FC6 | -0.01 | 0.01 | -0.03 | 0.01 | -0.05 |
| SELF-ESTEEM🡪FC6 | 0.03 | 0.02 | -0.01 | 0.08 | 0.11 |
| FCBASELINE🡪FC6 | -0.02 | 0.10 | -0.22 | 0.16 | -0.02 |
| FC5🡪CS6 | -0.54 | 0.22 | -0.97 | -0.08 | -0.33 |
| CS5🡪CS6 | 1.14 | 0.14 | 0.91 | 1.45 | 1.14 |
| FC6🡪CS6 | 0.58 | 0.19 | 0.25 | 0.95 | 0.34 |
| SCINT🡪CS6 | 0.21 | 0.35 | -0.49 | 0.87 | 0.06 |
| WC🡪CS6 | 0.32 | 0.18 | -0.03 | 0.68 | 0.25 |
| WC(QUAD)🡪CS6 | 0.05 | 0.07 | -0.10 | 0.18 | 0.07 |
| AGE🡪CS6 | -0.04 | 0.03 | -0.10 | 0.01 | -0.15 |
| DEP🡪CS6 | 0.00 | 0.02 | -0.04 | 0.03 | -0.01 |
| IMP SHAPE🡪CS6 | -0.15 | 0.15 | -0.47 | 0.15 | -0.13 |
| FCBASELINE🡪CS6 | -0.27 | 0.12 | -0.52 | -0.06 | -0.19 |
| Var(e.FC1) | 0.41 | 0.06 | 0.33 | 0.57 | 0.31 |
| Var(e.CS1) | 1.10 | 0.20 | 0.80 | 1.57 | 0.35 |
| Var(e.FC2) | 0.61 | 0.10 | 0.45 | 0.90 | 0.51 |
| Var(e.CS2) | 1.30 | 0.17 | 1.09 | 1.91 | 0.41 |
| Var(e.FC3) | 0.58 | 0.08 | 0.46 | 0.86 | 0.47 |
| Var(e.CS3) | 1.67 | 0.34 | 1.21 | 2.83 | 0.52 |
| Var(e.FC4) | 0.71 | 0.13 | 0.51 | 1.05 | 0.56 |
| Var(e.CS4) | 1.88 | 0.43 | 1.37 | 4.00 | 0.56 |
| Var(e.FC5) | 0.67 | 0.14 | 0.45 | 1.09 | 0.52 |
| Var(e.CS5) | 1.44 | 0.22 | 1.16 | 2.44 | 0.42 |
| Var(e.FC6) | 0.52 | 0.15 | 0.35 | 1.14 | 0.44 |
| Var(e.CS6) | 1.31 | 0.37 | 0.84 | 2.87 | 0.39 |
| Cov(e.FC1*e.FC2) | -0.16 | 0.06 | -0.32 | -0.07 | -0.32 |
| Cov(e.CS1*e.CS2) | -0.33 | 0.16 | -0.79 | -0.07 | -0.28 |
| Cov(e.FC2*e.FC3) | -0.18 | 0.09 | -0.40 | -0.03 | -0.30 |
| Cov(e.CS2*e.CS3) | -0.81 | 0.25 | -1.51 | -0.43 | -0.55 |
| Cov(e.FC3*e.FC4) | -0.29 | 0.09 | -0.61 | -0.17 | -0.46 |
| Cov(e.CS3*e.CS4) | -1.00 | 0.28 | -1.96 | -0.62 | -0.56 |
| Cov(e.FC4*e.FC5) | -0.26 | 0.13 | -0.60 | -0.07 | -0.38 |
| Cov(e.CS4*e.CS5) | -0.52 | 0.18 | -1.08 | -0.28 | -0.32 |
| Cov(e.FC5*e.FC6) | -0.30 | 0.12 | -0.76 | -0.14 | -0.51 |
| Cov(e.CS5*e.CS6) | -0.83 | 0.26 | -1.61 | -0.44 | -0.61 |
| Cov(FC0* CS0) | 0.88 | 0.17 | 0.54 | 1.23 | 0.44 |
| Cov(FC0*SCINT) | 0.01 | 0.05 | -0.09 | 0.12 | 0.02 |
| Cov(FC0*WC) | 0.43 | 0.14 | 0.17 | 0.71 | 0.26 |
| Cov(FC0*EDDUR) | -1.47 | 0.91 | -3.36 | 0.21 | -0.14 |
| Cov(FC0*SELF-ESTEEM) | -0.86 | 0.38 | -1.69 | -0.19 | -0.20 |
| Cov(FC0*FCBASELINE) | 0.80 | 0.13 | 0.56 | 1.06 | 0.55 |
| Cov(FC0*WC(QUAD)) | -0.76 | 0.28 | -1.38 | -0.26 | -0.23 |
| Cov(FC0*AGE) | -0.87 | 0.80 | -2.44 | 0.73 | -0.10 |
| Cov(FC0*DEP) | 2.86 | 1.08 | 0.76 | 4.95 | 0.22 |
| Cov(FC0*IMP SHAPE) | 0.25 | 0.16 | -0.09 | 0.56 | 0.13 |
| Cov(FC0*CSBASELINE) | 0.26 | 0.14 | 0.00 | 0.52 | 0.18 |
| Cov(CS0*SCINT) | -0.01 | 0.08 | -0.17 | 0.13 | -0.02 |
| Cov(CS0*WC) | 0.84 | 0.25 | 0.35 | 1.34 | 0.35 |
| Cov(CS0*EDDUR) | 2.84 | 1.32 | 0.37 | 5.58 | 0.19 |
| Cov(CS0*SELF-ESTEEM) | -1.05 | 0.53 | -2.11 | -0.01 | -0.17 |
| Cov(CS0*FCBASELINE) | 0.28 | 0.20 | -0.10 | 0.64 | 0.13 |
| Cov(CS0*WC(QUAD)) | -1.58 | 0.52 | -2.62 | -0.60 | -0.33 |
| Cov(CS0*AGE) | 2.78 | 1.17 | 0.34 | 5.00 | 0.21 |
| Cov(CS0*DEP) | 3.83 | 1.66 | 0.72 | 7.04 | 0.21 |
| Cov(CS0*IMP SHAPE) | 0.42 | 0.26 | -0.06 | 0.97 | 0.15 |
| Cov(CS0*FCBASELINE | 0.92 | 0.27 | 0.45 | 1.56 | 0.43 |
| Cov(SCINT*WC) | 0.01 | 0.06 | -0.10 | 0.12 | 0.01 |
| Cov(SCINT*EDDUR) | -0.46 | 0.36 | -1.17 | 0.23 | -0.11 |
| Cov(SCINT*SELF-ESTEEM) | 0.18 | 0.16 | -0.15 | 0.47 | 0.10 |
| Cov(SCINT*FCBASELINE) | 0.03 | 0.05 | -0.07 | 0.14 | 0.05 |
| Cov(SCINT*WC(QUAD)) | -0.14 | 0.11 | -0.37 | 0.07 | -0.10 |
| Cov( SCINT *AGE) | -0.21 | 0.32 | -0.82 | 0.41 | -0.06 |
| Cov( SCINT *DEP) | -0.61 | 0.46 | -1.47 | 0.35 | -0.12 |
| Cov(SCINT*IMP SHAPE) | 0.08 | 0.06 | -0.05 | 0.21 | 0.10 |
| Cov(SCINT*CSBASELINE | -0.06 | 0.06 | -0.19 | 0.04 | -0.10 |
| Cov(WC*EDDUR) | 1.17 | 1.15 | -1.21 | 3.31 | 0.10 |
| Cov(WC*SELF-ESTEEM) | -0.94 | 0.53 | -2.01 | -0.04 | -0.19 |
| Cov(WC*FCBASELINE) | 0.47 | 0.16 | 0.15 | 0.79 | 0.27 |
| Cov(WC*WC(QUAD)) | -2.27 | 0.68 | -3.73 | -1.09 | -0.57 |
| Cov(WC*AGE) | 0.45 | 1.10 | -1.96 | 2.42 | 0.04 |
| Cov(WC*DEP) | 4.72 | 1.47 | 1.95 | 7.52 | 0.31 |
| Cov(WC*IMP SHAPE) | 1.67 | 0.30 | 1.07 | 2.25 | 0.73 |
| Cov(WC*CSBASELINE) | 0.95 | 0.20 | 0.59 | 1.40 | 0.54 |
| Cov(WC*SELF-ESTEEM) | 1.59 | 2.31 | -2.71 | 6.41 | 0.05 |
| Cov(EDDUR*FCBASELINE) | -2.81 | 1.03 | -4.92 | -0.89 | -0.26 |
| Cov(EDDUR*WC(QUAD)) | 1.30 | 2.40 | -2.25 | 7.75 | 0.05 |
| Cov(EDDUR*AGE) | 58.08 | 12.63 | 36.45 | 86.77 | 0.88 |
| Cov(EDDUR*DEP) | 12.67 | 8.67 | -4.30 | 30.44 | 0.14 |
| Cov(EDDUR*IMP SHAPE) | 1.37 | 1.31 | -1.28 | 3.88 | 0.10 |
| Cov(EDDUR*CSBASELINE) | 1.41 | 0.75 | 0.05 | 3.10 | 0.13 |
| Cov(SELF-ESTEEM *FCBASELINE) | -1.14 | 0.39 | -1.90 | -0.35 | -0.25 |
| Cov(SELF-ESTEEM *WC(QUAD)) | 1.76 | 1.14 | -0.21 | 4.15 | 0.17 |
| Cov(SELF-ESTEEM *AGE) | 2.03 | 2.08 | -2.12 | 5.91 | 0.07 |
| Cov(SELF-ESTEEM *DEP) | -11.98 | 3.27 | -18.83 | -6.19 | -0.31 |
| Cov(SELF-ESTEEM *IMP SHAPE) | -0.81 | 0.71 | -2.32 | 0.46 | -0.14 |
| Cov(SELF-ESTEEM *CSBASELINE) | -0.47 | 0.39 | -1.20 | 0.35 | -0.10 |
| Cov( FCBASELINE*WC(QUAD)) | -0.91 | 0.31 | -1.58 | -0.34 | -0.26 |
| Cov( FCBASELINE*AGE) | -2.37 | 0.91 | -4.16 | -0.49 | -0.25 |
| Cov(FCBASELINE*DEP) | 1.03 | 1.14 | -1.22 | 3.15 | 0.08 |
| Cov(FCBASELINE*IMP SHAPE) | 0.37 | 0.17 | 0.04 | 0.72 | 0.19 |
| Cov(FCBASELINE *CSBASELINE) | 0.49 | 0.13 | 0.23 | 0.77 | 0.31 |
| Cov(WC(QUAD)*AGE) | 2.25 | 2.41 | -1.22 | 8.61 | 0.10 |
| Cov(WC(QUAD)*DEP) | -2.79 | 3.14 | -9.51 | 2.56 | -0.09 |
| Cov(WC(QUAD)*IMP SHAPE) | -2.68 | 0.79 | -4.35 | -1.21 | -0.59 |
| Cov(WC(QUAD)*CSBASELINE) | -1.29 | 0.41 | -2.12 | -0.53 | -0.37 |
| Cov(AGE*DEP) | 13.27 | 7.79 | -3.68 | 28.67 | 0.16 |
| Cov(AGE*IMP SHAPE) | 0.84 | 1.19 | -2.00 | 2.89 | 0.07 |
| Cov(AGE*CSBASELINE | 1.24 | 0.67 | -0.01 | 2.68 | 0.13 |
| Cov(DEP*IMP SHAPE) | 3.88 | 1.76 | 0.79 | 7.63 | 0.22 |
| Cov(DEP*CCBASELINE) | 2.84 | 1.15 | 0.79 | 5.28 | 0.21 |
| Cov(IMP SHAPE *CSBASELINE | 0.68 | 0.21 | 0.33 | 1.16 | 0.34 |
| Successful bootstraps | 986/1000 | | | | |

*Notes.* B=unstandardized coefficient. SE=standard error. β=standardised coefficient. CI=confidence interval. FC# = shape checking, where # indicates intervention week. SC# = concern about shape, where # indicates intervention week. SCINT = intervention status (shape checking intervention group vs. control). WC = weight concern at baseline. EDDUR = eating disorder duration at baseline. FCBASELINE = shape checking at baseline. AGE = age at baseline. DEP = depression at baseline. IMP SHAPE= importance of shape at baseline. CSBASELINE = concern about shape at baseline.

# **Hypothesis 4: Interpersonal Problem Severity as a Mediator of the Effect of Interpersonal Psychotherapy for Eating Disorders on Eating Disorder Psychopathology**

## Table 10: Multi-level model of the impact of IPT-ED on the purported mediator (interpersonal problem severity)

| **Model Components** | **Variable Details** | **B** | **Boot. SE** | **p** | **Bias-corrected 95% CI of B** | |
| --- | --- | --- | --- | --- | --- | --- |
|  |  |  |  |  | Lower | Upper |
| Baseline Predictors | Depression | 0.02 | 0.01 | .007 | 0.00 | 0.03 |
|  | Self-esteem | 0.02 | 0.02 | .425 | -0.03 | 0.06 |
|  | Self-esteem (quadratic trend) | -0.01 | 0.00 | .013 | -0.02 | -0.00 |
|  | Eating disorder duration | 0.02 | 0.01 | .054 | 0.00 | 0.04 |
|  | Eating disorder diagnosis (BN vs. EDNOS) | 0.35 | 0.17 | .037 | 0.02 | 0.68 |
|  | IPT-ED condition | 0.18 | 0.20 | .363 | -0.23 | 0.55 |
| Impact of Time | Time (linear trend) | -2.02 | 0.21 | .000 | -2.41 | -1.61 |
|  | Time (quadratic) | 0.40 | 0.06 | .000 | 0.27 | 0.52 |
| Impact of treatment allocation | IPT-ED condition*Time (linear trend) | -0.01 | 0.13 | .933 | -0.27 | 0.28 |
| Constant | Patients with average weight concern and eating disorder duration in CBT-E at baseline | 3.69 | 0.16 | .000 | 3.40 | 4.00 |
| Random effects  (log values) | SD (constant) | -0.27 | 0.08 | .001 | -0.48 | -0.21 |
|  | SD (residual) | 0.23 | 0.05 | .000 | 0.21 | 0.30 |
| Statistics | AIC | 1457.12 | | | | |
|  | Number of patients | 119 | | | | |
|  | Number of observations | 396 | | | | |
|  | Number of bootstraps | 1000/1000 | | | | |

*Notes.* B=unstandardized coefficient, *p*-value based on 95% confidence interval, CI=confidence interval. BN=bulimia nervosa. EDNOS=eating disorder not otherwise specified.

## Table 11: Multi-level model of the impact of IPT-ED on the outcome (eating disorder psychopathology)

| **Model Components** | **Variable Details** | **B** | **Boot. SE** | **p** | **Bias-corrected 95% CI of B** | |
| --- | --- | --- | --- | --- | --- | --- |
|  |  |  |  |  | Lower | Upper |
| Baseline Predictors | Eating disorder duration | 0.00 | 0.01 | .715 | -0.01 | 0.02 |
|  | Depression | 0.03 | 0.01 | .000 | 0.01 | 0.04 |
|  | IPT-ED condition | -0.02 | 0.16 | .919 | -0.33 | 0.29 |
| Impact of Time | Time (linear trend) | -1.92 | 0.20 | .000 | -2.30 | -1.51 |
|  | Time (quadratic) | 0.44 | 0.06 | .000 | 0.32 | 0.55 |
| Impact of baseline predictors during treatment | Eating disorder duration*Time (linear trend) | 0.01 | 0.00 | .009 | 0.00 | 0.02 |
| Impact of treatment allocation | IPT-ED condition*Time (linear trend) | 0.59 | 0.28 | .034 | 0.07 | 1.12 |
|  | IPT-ED condition*Time (quadratic trend) | -0.16 | 0.09 | .060 | -0.34 | -0.01 |
| Constant | Patients with average weight concern and eating disorder duration in CBT-E at baseline | 3.49 | 0.11 | .000 | 3.24 | 3.69 |
| Random effects  (log values) | SD (constant) | -0.30 | 0.07 | .000 | -0.51 | -0.24 |
|  | SD (residual) | -0.11 | 0.05 | .024 | -0.12 | -0.05 |
| Statistics | AIC | 1391.90 | | | | |
|  | Number of patients | 128 | | | | |
|  | Number of observations | 450 | | | | |
|  | Number of bootstraps | 1000/1000 | | | | |

*Notes.* B=unstandardized coefficient, *p*-value based on 95% confidence interval, CI=confidence interval.

## Table 12: Autoregressive partial mediation model of the relationships IPT-ED, interpersonal problem severity at the previous time point and within the same time point, and eating disorder psychopathology using full information maximum likelihood estimation for missing data

| **Model Components** | **B** | **Boot.**  **SE** | **Bias-corrected 95% CI of B** | | **β** |
| --- | --- | --- | --- | --- | --- |
|  |  |  | Lower | Upper |  |
| IPS0🡪IPS1 | 0.48 | 0.15 | 0.16 | 0.77 | 0.30 |
| Treatment (IPT-ED vs. CBT-E)🡪IPS1 | -0.22 | 0.36 | -0.91 | 0.50 | -0.06 |
| ED duration🡪 IPS1 | 0.02 | 0.02 | -0.02 | 0.05 | 0.08 |
| Depression🡪IPS1 | 0.00 | 0.02 | -0.03 | 0.04 | 0.01 |
| ED diagnosis🡪 IPS1 | 0.40 | 0.37 | -0.29 | 1.17 | 0.11 |
| Self-esteem🡪 IPS1 | 0.01 | 0.05 | -0.08 | 0.11 | 0.03 |
| Self-esteem (quadratic trend)🡪 IPS1 | -0.02 | 0.01 | -0.03 | -0.00 | -0.20 |
| IPS1🡪EDP1 | 0.25 | 0.07 | 0.10 | 0.37 | 0.34 |
| IPS0🡪EDP1 | -0.10 | 0.10 | -0.28 | 0.11 | -0.08 |
| EDP0🡪EDP1 | 0.46 | 0.11 | 0.23 | 0.67 | 0.36 |
| Treatment (IPT-ED vs. CBT-E)🡪EDP1 | 0.83 | 0.23 | 0.36 | 1.28 | 0.32 |
| Eating disorder duration🡪EDP1 | 0.00 | 0.01 | -0.02 | 0.03 | 0.02 |
| Depression🡪EDP1 | 0.00 | 0.01 | -0.02 | 0.02 | 0.01 |
| IPS1🡪IPS2 | 0.31 | 0.34 | -0.44 | 0.94 | 0.35 |
| Treatment (IPT-ED vs. CBT-E)🡪IPS2 | 0.18 | 0.32 | -0.43 | 0.82 | 0.06 |
| ED duration🡪 IPS2 | 0.02 | 0.02 | -0.01 | 0.06 | 0.10 |
| Depression🡪IPS2 | 0.01 | 0.02 | -0.03 | 0.04 | 0.04 |
| ED diagnosis🡪 IPS2 | 0.42 | 0.39 | -0.28 | 1.26 | 0.13 |
| Self-esteem🡪 IPS2 | 0.02 | 0.05 | -0.06 | 0.11 | 0.05 |
| Self-esteem (quadratic trend)🡪 IPS2 | -0.01 | 0.01 | -0.03 | 0.02 | -0.07 |
| IPS1🡪EDP2 | -0.17 | 0.10 | -0.39 | 0.04 | -0.23 |
| EDP1🡪EDP2 | 0.94 | 0.35 | 0.38 | 1.84 | 0.95 |
| IPS2🡪EDP2 | 0.18 | 0.09 | 0.00 | 0.35 | 0.22 |
| Treatment (IPT-ED vs. CBT-E)🡪EDP2 | -0.53 | 0.38 | -1.46 | 0.06 | -0.20 |
| Eating disorder duration🡪EDP2 | 0.01 | 0.01 | -0.01 | 0.04 | 0.08 |
| Depression🡪EDP2 | 0.00 | 0.01 | -0.02 | 0.03 | 0.03 |
| IPS2🡪IPS3 | 0.73 | 0.64 | 0.24 | 1.50 | 0.76 |
| Treatment (IPT-ED vs. CBT-E)🡪IPS3 | -0.16 | 0.36 | -0.88 | 0.42 | -0.05 |
| ED duration🡪 IPS3 | 0.02 | 0.02 | -0.02 | 0.06 | 0.14 |
| Depression🡪IPS3 | 0.01 | 0.01 | -0.02 | 0.03 | 0.07 |
| ED diagnosis🡪 IPS3 | -0.46 | 0.40 | -1.26 | 0.15 | -0.15 |
| Self-esteem🡪 IPS3 | 0.04 | 0.05 | -0.06 | 0.13 | 0.09 |
| Self-esteem (quadratic trend)🡪 IPS3 | -0.01 | 0.01 | -0.03 | 0.01 | -0.16 |
| IPS2🡪EDP3 | 0.02 | 0.20 | -0.20 | 0.25 | 0.02 |
| EDP2🡪EDP3 | 0.58 | 0.58 | 0.24 | 0.99 | 0.62 |
| IPS3🡪EDP3 | 0.15 | 0.10 | -0.02 | 0.37 | 0.19 |
| Treatment (IPT-ED vs. CBT-E)🡪EDP3 | 0.27 | 0.19 | -0.10 | 0.64 | 0.11 |
| Eating disorder duration🡪EDP3 | 0.02 | 0.02 | -0.00 | 0.04 | 0.12 |
| Depression🡪EDP3 | 0.01 | 0.02 | -0.01 | 0.03 | 0.07 |
| var(e.IPS1) | 2.77 | 0.28 | 2.42 | 4.01 | 0.85 |
| var(e.EDP1) | 1.15 | 0.15 | 0.93 | 1.54 | 0.66 |
| var(e.IPS2) | 1.96 | 0.81 | 1.27 | 4.11 | 0.78 |
| var(e.EDP2) | 1.18 | 0.51 | 0.77 | 2.95 | 0.69 |
| var(e.IPS3) | 1.49 | 15.43 | 0.85 | 4.85 | 0.65 |
| var(e.EDP3) | 0.68 | 8.52 | 0.45 | 1.34 | 0.45 |
| cov(e.IPS1*e.IPS2) | 0.12 | 0.89 | -1.45 | 2.08 | 0.05 |
| cov(e.EDP1*e.EDP2) | -0.47 | 0.36 | -1.37 | 0.08 | -0.41 |
| cov(e.IPS2*e.IPS3) | -0.51 | 1.24 | -2.25 | 0.50 | -0.30 |
| cov(e.EDP2*e.EDP3) | -0.07 | 0.59 | -0.54 | 0.41 | -0.08 |
| cov(IPS0*EDP0) | 0.21 | 0.09 | 0.06 | 0.41 | 0.19 |
| cov(IPS0*IPT-ED condition) | 0.08 | 0.05 | -0.01 | 0.18 | 0.14 |
| cov(IPS0*eating disorder duration) | 0.79 | 0.91 | -1.15 | 2.38 | 0.08 |
| cov(IPS0*depression) | 4.03 | 1.12 | 2.05 | 6.33 | 0.33 |
| cov(IPS0*ED diagnosis) | 0.07 | 0.05 | -0.03 | 0.17 | 0.13 |
| cov(IPS0*self-esteem) | -0.38 | 0.41 | -1.29 | 0.34 | -0.09 |
| cov(IPS0*self-esteem (quadratic trend)) | 0.30 | 2.52 | -6.15 | 4.39 | 0.01 |
| cov(EDP0*IPT-ED condition) | -0.02 | 0.05 | -0.11 | 0.06 | -0.04 |
| cov(EDP0*eating disorder duration) | 0.72 | 0.87 | -1.27 | 2.24 | 0.08 |
| cov(EDP0*depression) | 4.32 | 1.12 | 2.36 | 6.62 | 0.39 |
| cov(EDP0*ED diagnosis) | 0.07 | 0.04 | -0.01 | 0.16 | 0.13 |
| cov(EDP0*self-esteem) | -0.78 | 0.38 | -1.68 | -0.15 | -0.21 |
| cov(EDP0*self-esteem (quadratic trend)) | -2.85 | 2.85 | -10.88 | 0.92 | -0.14 |
| cov(IPT-ED conditon*eating disorder duration) | 0.75 | 0.36 | 0.08 | 1.48 | 0.17 |
| cov(IPT-ED condition*depression) | 0.39 | 0.47 | -0.61 | 1.25 | 0.07 |
| cov(IPT-ED condition*ED diagnosis) | 0.01 | 0.02 | -0.03 | 0.05 | 0.05 |
| cov(IPT-ED condition*self-esteem) | -0.24 | 0.16 | -0.54 | 0.09 | -0.13 |
| cov(IPT-ED condition*self-esteem (quadratic trend)) | -0.06 | 0.89 | -1.58 | 1.94 | -0.01 |
| cov(eating disorder duration*depression) | 12.79 | 9.27 | -5.13 | 30.14 | 0.14 |
| cov(eating disorder duration*ED diagnosis) | 0.07 | 0.38 | -0.67 | 0.79 | 0.02 |
| cov(eating disorder duration*self-esteem) | 1.62 | 2.36 | -2.57 | 6.34 | 0.05 |
| cov(eating disorder duration*self-esteem (quadratic trend)) | 0.78 | 12.11 | -23.31 | 23.15 | 0.00 |
| cov(depression*ED diagnosis) | 0.61 | 0.48 | -0.32 | 1.62 | 0.11 |
| cov(depression*self-esteem) | -11.94 | 3.20 | -18.68 | -6.03 | -0.31 |
| cov(depression*self-esteem (quadratic trend)) | -32.36 | 18.65 | -80.71 | -4.27 | -0.15 |
| cov(ED diagnosis*self-esteem) | -0.14 | 0.17 | -0.50 | 0.17 | -0.08 |
| cov(ED diagnosis*self-esteem (quadratic trend)) | 0.26 | 0.86 | -1.41 | 2.02 | 0.03 |
| cov(self-esteem*self-esteem (quadratic trend)) | 31.24 | 14.79 | 7.99 | 65.69 | 0.45 |
| Successful Bootstraps | 883/1000 | | | | |

*Notes.* B=unstandardized coefficient. SE=standard error. β=standardised coefficient. CI=confidence interval. IPS# = interpersonal problem severity, where # indicates time point (0 = baseline, 1 = end of treatment, 2 = 20-week follow-up, 3 = 40-week follow-up. EDP# =eating disorder psychopathology, where # indicates time point. ED=eating disorder. Eating disorder duration, depression and self-esteem measured at baseline.
